# Supplementary material for: In-silico simulated prototype-patients using TPMS technology to study a potential adverse effect of sacubitril and valsartan
Source: PLoS One. 2020 Feb 13;15(2):e0228926. doi: 10.1371/journal.pone.0228926 (PMC7018085; doi:10.1371/journal.pone.0228926)
Supplement: S1 File — (DOCX) [file pone.0228926.s001.docx]

**Supplementary material for:**

**In-silico simulated prototype-patients using TPMS technology to study a potential adverse effect of sacubitril and valsartan**

Guillem Jorba, Joaquim Aguirre-Plans, Valentin Junet, Cristina Segú-Vergés, José Luis Ruiz, Albert Pujol, Narcis Fernandez-Fuentes, José Manuel Mas and Baldo Oliva

[**Extended version of materials and methods 2**](#_Toc27735438)

[**Extended version of results and discussion 8**](#_Toc27735439)

[**Supplementary Figures 14**](#_Toc27735440)

[**Supplementary Tables 17**](#_Toc27735441)

[**References 25**](#_Toc27735442)

Extended version of materials and methods

**1. Biological Effectors Database (BED) to molecularly describe specific clinical conditions**

Patient-like characteristics are modelled using clinical data and/or experimental molecular data. There are many databases providing clinical data of patients, adverse drug reactions, diseases or indications (e.g. ClinicalTrials.gov, SIDER, ChEMBL, PubChem, DrugBank…). Many other databases provide molecular data defining the existing human genes and/or proteins and describing the relationships between them (IntAct, BioGRID, REACTOME…). Combining both, clinical and molecular information available, the BED describes more than 300 clinical phenotypes as sets of genes and proteins (effectors) that can be “active”, “inactive” or “neutral” [1,2]. For example, in a metabolic protein-like network, an enzyme will become “active” in the presence of a catalyst, or become inactivated when interacting with an inhibitor. Alternatively, in a genetic network, genes are active when they are expressed (experimentally detected as over-expression) and inactive when they are repressed (experimentally detected as under-expression). Additionally, in protein-protein interaction (PPI) networks, some proteins carry out their interactions only when they are phosphorylated, thus becoming active, and vice versa by dephosphorylation. By default, neutral proteins remain unaffected, neither active nor inactive, for a particular phenotype.

The methodology used for assigning the protein effectors to each pathology starts by defining the pathophysiological processes (functions) according to the general definitions used by the scientists studying the disease. Then, a review of the most recent, relevant and accepted information in the field is performed through PubMed queries, starting from general pathophysiology reviews. An expansion of the effector candidate’s identification is done through reading the relevant original papers from the references or adding searches of important concepts that are not covered enough (molecularly wise) within the reviews read. The final goal of the characterization is to select proteins with an accepted functional role within the disease, and specifically within the functions that define the disease to center the analysis.

**1.1. HF effectors**

Regarding the molecular basis of HF BED proteins, they were characterized as described above and in *Iborra-Egea et al. (2017)* [3]. The definition used of heart failure in the current study has been performed according to the indication of Entresto and to the EMA Assessment report [4]. Thus, it is centered in processes associated to long term changes related to cardiac remodeling (as discussed in the paper were the models were initially presented [3]), that can be cause and consequence of heart failure, not necessarily caused by ischemic causes. The identified functions are detailed in Supplementary Table 12.

**1.2. MD effectors**

MD pathophysiology is tightly related to protein accumulation [5–7]. However, the characterization used for the current study not only included this function, but also other processes associated to MD pathophysiology, including neovascularization, characteristics of wet Age-Related MD and changes associated to geographic atrophy (late stage dry Age-Related MD) [8]. The functions are detailed in Supplementary Table 13.

**2. TPMS modelling**

The Therapeutic Performance Mapping System (TPMS) is a tool that creates mathematical models of a drug/pathology protein pathways to explain a clinical outcome or phenotype [2,3,9–13]. These models find MoAs that explain how a *Stimulus* (i.e. proteins activated or inhibited by a drug) produces a *Response* (i.e. proteins active or inhibited in a phenotype). As an example of usage, here we applied TPMS to the drug-indication pair sacubitril/valsartan and HF. Regarding the drug, we retrieved the sacubitril/valsartan targets from DrugBank [14], PubChem [15], STITCH [16], SuperTarget [17] and hand curated literature revision. As for the indication, we retrieved the proteins whose modulations had been associated with HF from the BED [1,2]. Finally, after applying the TPMS methodology, we obtained a set of connected proteins (subnetworks) with associated activities, each subnetwork with a potential explanation of the molecular mechanism of the drug in agreement with what had been previously described (i.e. a potential MoA).

**2.1. Building the Human protein network (HPN)**

To apply the TPMS approach and create the mathematical models of MoAs, an HPN is needed beforehand. In this study, we used a PPI network created from the integration of public and private databases: KEGG [18], BioGRID [19], IntAct [20], REACTOME [21], TRRUST [22], and HPRD [23]. In addition, information extracted from scientific literature, which was manually curated, was also included and used for trimming the network. The resulting HPN considers interactions corresponding to different tissues to take into account the effect of the *Stimulus* in the whole body.

**2.2. Defining model restrictions**

A collection of restrictions, defined as the true set of edges and nodes with the property of being active or inactive, are used for validating the models obtained with TPMS. We define two types of restrictions depending on its specificity. The general or global restrictions are those used in all approaches and describe a wide expanse of knowledge about protein interactions and relations. This information is obtained from HPRD [23], DIP [24], TRRUST [22], INTACT [20], REACTOME [21], BIOGRID [19], SIDER [25] and DrugBank [14]. These set of restrictions help indicate what proteins are active or inactive, and their interactions, in a general human being. Additionally, specific restrictions regarding the phenotype under study can also be used, usually derived from high throughput data or additional protein knowledge.

For this study, we added specific information to our models concerning the changes of gene expression induced by sacubitril/valsartan on HF patients. Specifically, we used the GSE57345 gene expression dataset [26] , extracted from GEO database, as in *Iborra-Egea et al. (2017)* [3]. We calculated the expression fold change of genes associated with the HPN and mapped them as activated or inhibited proteins (active if they corresponded to over-expressed genes and inactive -inhibited- for under-expressed).

**2.3. Description of the mathematical models**

The algorithm of TPMS for generating the models is similar to a Multilayer Perceptron of an Artificial Neural Network over the HPN (where neurons are the proteins and the edges of the network are used to transfer the information). It takes as input signals the activation (+1) and inactivation (-1) of the drug target proteins and as output the BED protein states of the pathology phenotype. The network is limited to only interactions that connect the drug targets with any other protein in the HPN in a maximum of three steps to avoid signal noisiness. Once set, the algorithm optimizes the paths between both input and output protein sets and computes the activation and inactivation values of the all proteins in the HPN. The parameters to solve are the weights associated to the links between every node pair ($\omega_{l}$). Each node of the protein network receives as input the output of the incoming connected nodes, which are weighted by each link weight. The sum of inputs is transformed by a hyperbolic tangent function to generate the score of the node (neuron), which become the “output signal” of the current node towards outgoing nodes. Details of the approach are shown in **Fig 1a**, where $n_{5}$ is linked to $n_{1}$ and $n_{2}$. The output signal of $n_{5}$ is $n_{5}=\tanh\left( n_{1}\cdot\omega_{1-5} + n_{2}\cdot\omega_{2-5} \right)$. The $\omega_{l}$ parameters are obtained by optimization, using a Stochastic Optimization Method based on Simulated Annealing [27], such that the values of the effector nodes are the closest to their expected values, and always adjusting to the maximum of the restrictions mentioned above. The iterative process of optimization usually requires between 10^6^ and 10^9^ iterations, until satisfying at least the 80% of the restrictions and the values of the effectors. However, the number of $\omega_{l}$ parameters can be very high (between 100,000 and 400,000 depending on the size of the subnetwork) and the size of the collection of restrictions (approximately 10^7^) is usually not enough to find a unique solution. For that, many final models can be obtained and manual curation can be applied to select and modify the network and reduce the space of exploration.

**3. Measures to compare sets of MoAs**

TPMS returns a set of MoAs describing potential relationships between the targets of a drug and the biological protein effectors of a disease. We hypothesize that TPMS solutions represent MoAs in different prototype-patients. Therefore, we needed to define some comparison measures in order to understand the relationships between all potential mechanisms and compare sets of MoAs from different views.

**3.1. Intensity of the response**

We defined the “intensity” of the response as a pair: 1) the number of protein effectors (#) achieving an expected signal sign; and 2) a measure of the strength of the output signal of the effectors (i.e. a global measure of the output signal, named TSignal). For the present study, however, only the TSignal was used.

Assuming $y_{i}$ as the value achieved by a protein effector “i”, while $v_{i}$ is the effector sign according to the BED (active or inactive) and $n$ is the total number of effectors described for a phenotype, we define:

- **Number of effectors achieving the expected** **sign**: We expect that a drug will revert the conditions of a disease phenotype, while it may reach the effectors of an adverse event. Consequently, a drug should inactivate the active protein effectors of a pathology-phenotype and activate the inactive ones, but it could activate/inhibit other adverse event effectors with the same sign as described in the BED. Using Dirac’s δ (i.e. δ(0)=1, and zero otherwise), for drug indications the formula is defined as following:

${\#}_{indication} = \sum_{i=1}^{n} \delta\left( v_{i}+\frac{y_{i}}{\left| y_{i} \right|} \right)$ **[Equation 1a]**

Therefore, in the case of the disease effectors we only count the effectors with a BED value of opposite sign to the signal arriving from the drug.

However, for adverse events, the formula changes because we count the effectors that are affected by the drug, such that the signal arriving from the drug has the same sign as in the BED:

${\#}_{adverse event} = \sum_{i=1}^{n} \delta\left( v_{i}-\frac{y_{i}}{\left| y_{i} \right|} \right)$ **[Equation 1b]**

- **TSignal**: The average of the output values of the protein effectors such that the proteins with correct sign are considered as positive signal, and the ones with the incorrect sign considered as negative signal. For a drug affecting the phenotype of a disease, this implies that $v_{i}$ and $y_{i}$ have opposite sign and we need to change the sign:

${TSignal}_{indication}= -\frac{1}{n}\sum_{i=1}^{n} v_{i}y_{i}$ **[Equation 2a]**

On the contrary, to test if a drug induces an adverse event, we check if the output signal has the same sign as the effectors of the desired phenotype, and therefore TSignal is defined as:

${TSignal}_{adverse event}= \frac{1}{n}\sum_{i=1}^{n} v_{i}y_{i}$ **[Equation 2b]**

**3.2. Distance between two sets of MoAs**

We used the modified Hausdorff distance (MHD) introduced by Dubuisson and Jain [28] as the *distance* between two or more sets of MoAs in order to determine their similarity. We used the distance measures between two (finite) point sets A and B as following:

$$\text{For }a\in A, d\left( a,B \right):= \min_{b\in B} d\left( a,b \right),$$

$${\text{and }d}_{A}\left( B \right):= \frac{1}{\left| A \right|}\sum_{a\in A} d(a,B),$$

Where |A| is the number of elements in A, d(∙,∙) is the Euclidean distance and “a” and “b” are n-tuples of the activities (output signals) of the nodes of two MoAs (a in A and b in B). Then, we defined the MHD as:

$d_{\text{MHD}}\left( A,B \right):=\text{max (} d_{A}\left( B \right),d_{B}\left( A \right) )$ **[Equation 3]**

Note that the MHD is a semimetric and not a metric, since the triangular inequality does not hold.

**3.3. Potential biomarkers extracted from MoAs**

***3.3.1. Identification of Best-Classifier Proteins***

In order to extract potential biomarkers from comparing sets of MoAs, we first defined the *best-classifier proteins*, specific proteins helping us to infer biological associations and distinguish the responses of drugs on a population (i.e. potential biomarkers). Best-classifier proteins (single or pairs) are the proteins inside the HPN that allow to better classify samples between groups of MoAs. These classifiers are determined by a Data-Science strategy, which is based on a set of Feature Selection algorithms combined with several Base Classifiers. The feature selection used for single proteins was brute force [29], so analyzing one feature or protein at a time, while for protein pairs the following selection methods were used: elastic net [30]; entropy and correlation [31]; LASSO [32]; random forest [33]; GLM random sets [34]; ReliefF [35]; Ridge regression [36]; simple regression [37]; Wilcoxon test [38]; and Wilcoxon test with correlation [38]. Several base classifiers were applied to distinguish the two groups using the selected features: optimal threshold; linear regression [37]; Multilayer Perceptron Network [39]; Generalized Linear Model [34]; elastic net [40]; and optimal quadratic threshold [41]. Finally, after a k-fold cross-validation (k=10) [42] was applied, the proteins were sorted by the balanced accuracy [43] of the classification. For this study, only the 200 proteins (or pair of proteins) with highest balanced accuracy were selected as best-classifier proteins. Assuming the hypothesis that the selected MoAs are representative of individual prototype-patients, these proteins could then be used as biomarkers to classify a cohort of patients by the activity or absence of activity of the proteins.

***3.3.2. Identification of differential Best-Classifier Proteins***

Each best-classifier protein has a specific distribution of signal values corresponding to each group of MoAs. We applied the Mann-Whitney *U* test to compare the two distributions and selected those proteins having a significantly different distribution (p-value< 0.01). We also restricted the list to proteins having an average value with opposite sign among groups (i.e. positive vs. negative or vice versa), and named them as *differential best-classifier proteins*. By following this strategy, we can identify two groups of differential best-classifier proteins: those active in the first group (positive output signal in average) and inactive in the other (negative output signal in average), and the opposite.

***3.3.3. Types of proteins not considered***

- **Non-differential Best-Classifier Proteins**: Those are proteins in which, even if the mean signal in both groups is very similar, the machine learning algorithms are still able to differentiate High- and Low- MoAs based on their distribution values. For example, in the upper right corner of Figure 2a we find the protein P29353, the 181st best protein to classify High- and Low- HF models (cross-validation AUC = 0.67, P-value = 1.12·10-4). P29353 has a Low-HF mean signal of 0.99999999948 and a High-HF mean signal of 0.9999999985. As showed in **Supplementary Fig 4a**, the High- and Low- HF signals values are both very close to each other. However, if we explore the distribution of signals considering all the decimals given by TPMS (**Supplementary Fig 4b**), we can observe a slight difference between the two distributions. This fact allowed the machine learning algorithms to include the protein as a best-classifier protein, but was then rejected as a differential best-classifier protein after applying the Mann-Whitney *U* test.

- **Differential non-Best-Classifier Proteins**: Those are proteins that, when comparing the signals between groups, they have significantly opposite sign. However, they are not considered Best-Classifier Proteins because they are not among the top 200 proteins selected by the machine learning algorithms. For example, the protein P40763 is the 241st best feature on distinguishing High- and Low- Heart Failure Mechanisms of Action (cross-validation AUC = 0.66, P-value = 1.22·10^-3^). The distribution of High- and Low signals are represented in **Supplementary Fig 5**. In the figure we can appreciate how the distributions of High- and Low- signals are overlapped, complicating their differentiation. Still, the p-value of the cross-validation is below 0.05, reflecting the potential of this feature to differentiate the distinct types of Mechanisms of Action.

Extended version of results and discussion

We applied TPMS to the HPN using as input signals the drug targets of sacubitril/valsartan (NEP / AT1R) and as output signals the proteins associated with HF extracted from the BED. Out of all MoAs found by TPMS, we selected the 200 satisfying the largest number of restrictions (and at least 80% of them) to perform further analysis.

Note that TPMS was only executed once, optimizing the results to satisfy the restrictions on HF data. The values of MD are obtained by measuring the signal arriving at the MD effectors, which are part of the HPN and also receive signal. This procedure was chosen because we defined HF as the indication of the drug (sacubitril/valsartan), while MD is a potential adverse effect.

**1. Stratification of MoAs**

In order to compare models related to a good or bad response to the treatment, or those more prone to lead towards potential MD adverse effect, we stratified the MoAs. For HF, or treatment response, MoAs were ranked by their TSignal and then split in four quartiles. The first quartile (top 25%) contains MoAs with higher intensity of the response, which in turn corresponds to lower values of the effectors associated with HF phenotype (we named them as “Low”-disease MoAs). On the contrary, the fourth quartile (bottom 25%) collects MoAs with lower intensity of response (thus, we named as “High”-disease MoAs) (**Supplementary Fig 1a**). On the other hand, for MD, the first quartile (top 25%) contains MoAs with higher intensity, which as an adverse event, correspond to models with high values of the effectors associated to MD (we named them as High-adverseEvent MoAs). The fourth quartile (bottom 25%) collects MoAs with lower intensity of response (thus, we named as Low- adverseEvent MoAs) (**Supplementary Fig 1b**). Note that, in the following steps and because HF and MD groups were extracted from the same 200 set of models, common MoAs between different HF and MD-defined sets could be expected.

**2. Comparison of MoAs with high/low TSignal associated to HF or MD**

We calculated the modified Hausdorff distance between the groups of MoAs (High-MD, Low-MD, High-HF and Low-HF) to elucidate their similarity values (**Supplementary Table 5**). In this sense, the higher distance between the groups, the more different they are. We used these distances to calculate a dendrogram tree (see **Supplementary Fig 2**) showing that MoAs associated with a bad response to sacubitril/valsartan for HF (high-HF) are more similar (i.e. closer) to MoAs linked to a stronger MD adverse effect (high-MD). It is remarkable that the distances between Low-HF and High-HF and between Low-MD and High-MD are larger than the cross distances between HF and MD. However, by the definition of distance (equation 3 in supplementary material), we cannot account for the dispersion among the MoAs within and between each group. Therefore, for each set we calculated the mean Euclidean distance between all the points and its center, defined by the average of all points (see **Supplementary Table 6**). As a result, all groups showed very similar dispersion values.

In order to have a global and graphical view of the distance between the individual MoAs, we generated a multidimensional scaling (MDS) plot calculated using MATLAB (see **Fig 2**). MDS plots display the pairwise distances in two dimensions while preserving the clustering characteristics (i.e. close MoAs are also close in the 2D-plot and far MoAs are also far in 2D). Focusing on the Low-HF group depicted in blue circles, we observe that there is no clear tendency to cluster with any of the MD groups. There are few cases of Low-HF MoAs coinciding in the space with Low- or High-MD MoAs. This implies that a good response to sacubitril/valsartan of HF patients would not be usually linked to the development of MD. Moreover, no clear distinction is found when plotting only the MD MoAs within the Low-HF group (see **Supplementary Fig 3a**). However, regarding the set of High-HF MoAs, we can differentiate two clusters of MoAs: one related to the High-MD group (green crosses); and the other close to MoAs of the Low-MD group (black crosses) (see **Supplementary Fig 3b**).

Assuming the hypothesis that different MoAs correspond to distinct prototype-patients, we conclude that for the specific set of patients for which sacubitril/valsartan works best reducing HF, it would be more difficult to differentiate between those presenting MD and those who do not. Instead, for the High-HF group, patients having MD could indeed be easily distinguished from those not presenting MD as side effect. However, because Low-HF group has more relevance to the clinics, specific functional analyses were performed in this specific group, as seen in following sections.

**3. Identification and functional analysis of potential biomarkers**

For this section, we identified the nodes (i.e. proteins) significantly differentiating two groups of models (using a Mann-Whitney *U* test) for which the average of output signals have opposite signs (see methods in 3.3). After that, the function of the identified proteins was extracted from Gene Ontology (GO).

**3.1. Identification of best-classifier proteins differentiating HF responses**

After the model stratification regarding the HF groups, we selected the 200 best-classifier proteins to differentiate the two groups of MoAs. Among these proteins, we identified the differential best-classifier proteins as explained in the methodology, and ended up with two groups: those active in Low-HF (the average of output signals in Low-HF MoAs is positive) and inactive in High-HF (the average of output signals in High-HF MoAs is negative); and those active in High-HF but inactive in Low-HF. Out of the starting 200 best-classifier proteins, we found a total of 45 differential best-classifier proteins associated with the treatment response (6 in the first group and 39 in the second) (see **Supplementary Table 1**). **Fig 3a** displays all the proteins average signal values for the MoAs of Low-HF vs High-HF. Most of the proteins with opposite signs between the two cohorts were also selected as differential best-classifier proteins.

To pinpoint the biological role of these proteins, we first identified the GO enriched functions (see **Supplementary Table 2)** and then searched in the literature for evidences linking them with HF. The enrichment used for this proceeding was calculated using the software FuncAssociate [44]. Among the enriched functions, we found processes associated with the SCAR complex, the positive regulation of actin nucleation, the regulation of neurotrophin TRK receptor and dendrite extension. We used the same procedure to extract the GO functions associated to the differential best-classifier proteins that are inactive in Low-HF but active in High-HF. We detected functions such as phosphatidylinositol kinase activity, MAP kinase activity, DNA damage induced protein phosphorylation and superoxide anion generation. Although some enriched functions are shared by both sets, such as Fc gamma receptor signaling, the majority of functions identified are different (see **Supplementary Table 2)**.

Some of the proteins and functions highlighted in the current analysis have been related to myocardial function. On the one hand, our findings show that differential best-classifier proteins Low-HF-active/High-HF-inactive point towards an important role for actin nucleation and polymerization mechanisms in drug response (reflected by the functions *regulation of actin nucleation*, *regulation of Arp2/3 complex-mediated actin nucleation*, *SCAR complex*, *filopodium tip*, or *dendrite extension*). In fact, the alteration of actin nucleation and polymerization mechanisms has been reported in heart failure [45–47]. Interestingly, a role for the activation of another differential best-classifier candidate, ATGR2, has been proposed to mediate some of the beneficial effects of angiotensin II receptor type 1 antagonists, such as valsartan [48,49].

On the other hand, the results of the differential best-classifier proteins Low-HF-inactive/High-HF-active are linked to phosphatidylinositol kinase mediated pathways (*phosphatidylinositol-3,4-bisphosphate 5-kinase activity*) and MAP kinase mediated pathways (*MAP kinase kinase activity*, best classifier proteins MAPK1, MAPK3, MAPK11, MAPK12 or MAPK13). In this case, both signaling pathways have been associated to cardiac hypertrophy and subsequent heart failure [50,51]. These outcomes clearly leads towards the idea that High-HF models are a representation of prototype-patients with a worst response to the treatment, while Low-HF models are related to more beneficial response to the medication.

**3.2. Identification of best-classifier proteins differentiating MD responses**

We similarly classified MoAs in High-MD and Low-MD identified the differential best-classifier proteins active in Low-MD but inactive in High-MD, and vice versa. As before, we compared the distributions of Low-MD and High-MD output signals of the best-classifier proteins and calculate the average of the signal in all MoAs in Low- and High- MD. Out of 200 best-classifier proteins, we identified 28 Low-MD-active/High-MD-inactive and 29 Low-MD-inactive/High-MD-active (see **Supplementary Table 3**). **Fig 3b** shows the plot for all proteins classified by their average output signal in Low-MD and High-MD models.

Again, we calculated the GO enriched functions for these groups of proteins (see **Supplementary Table 4**). For the first group (Low-MD-active/High-MD-inactive) we obtained unique functions such as dendritic spine development, positive regulation of vascular endothelial growth factor production and phosphotyrosine binding. For the second group (Low-MD-inactive/High-MD-active), we found functions such as dorsal/ventral axon guidance, fibroblast growth factor receptor binding and response to toxic substance. However, phosphatidylinositol bisphosphate kinase activity showed up as enriched function in both groups.

Some of the proteins and functions underlined in the current analysis had previously been related to MD. The presence of dendritic spine development and dorsal/ventral axon guidance related proteins among the differential best-classifiers points towards a role for sacubitril/valsartan-associated MD in dendritic and synaptic plasticity mechanisms, which had been previously linked to the condition [52]. Furthermore, valsartan treatment has been reported to promote dendritic spine development in other related neurodegenerative diseases, such as Alzheimer’s disease [53]. Other functions enriched within the differential best-classifier proteins (Low-MD-inactive/High-MD-active) are implicated in growth factor related pathways, which are known to be involved in wet MD pathogenesis [54]. Moreover, neovascularization in the wet variant of MD has been linked to the signaling of some of the growth factors detected as sacubitril/valsartan-associated MD classifiers in this study, including FGF1 [54] and PDGF [55,56].

**3.3. Identification of potential biomarkers differentiating MD responses in Low-HF**

We previously mentioned that some MoAs could be shared between the different groups of HF and MD (**Supplementary Table 7**). Knowing that, we focused on the shared MoAs between Low-HF and High-MD to analyze the special case comprising prototype-patients in which the treatment best reduces HF disease but increases MD adverse effect. In order to identify these patients, we compared the Low-HF ∩ Low-MD with Low-HF ∩ High-MD MoAs; **Table 1** shows the 30 biomarkers identified. On the one hand, we found 16 proteins active in Low-HF ∩ Low-MD MoAs but inactive in Low-HF ∩ High-MD (15 of them shared with MD best-classifier proteins). On the other hand, 14 proteins were identified as inactive in Low-HF ∩ Low-MD and active in Low-HF ∩ High-MD MoAs (12 of them were MD best-classifier proteins). We calculated the GO enriched functions of these two groups and observed that “phosphatidylinositol bisphosphate kinase activity” is enriched among proteins that are active in Low-HF ∩ Low-MD MoAs. Instead, “fibrinolysis” was found to be enriched among proteins active in Low-HF ∩ High-MD MoAs (**Table 2**). With this, we conclude that among the group of prototype-patients for which sacubitril/valsartan improves HF treatment response, the modulation of fibrinolysis could play a role at inducing the MD adverse effect. Moreover, we propose 12 best-classifier proteins that may be considered as biomarkers for good prognosis of the side effect.

In fact, since neovascular MD development is characterized by subretinal extravasations of novel vessels derived from the choroid (CNV) and the subsequent hemorrhage into the photoreceptor cell layer in the macula region [8], it might be reasonable to think that the modulation of fibrinolysis and blood coagulation pathways could play a role. The reported implication of some fibrinolysis related classifiers, such as FGB, SERPINE1 (PAI-1), and SERPING1, in neovascular MD development seems to support this hypothesis [57–59]. Besides, valsartan might be implicated in this mechanism, since it has been reported to modulate PAI-1 levels and promote fibrinolysis in different animal and human models [60,61].

In addition, the presence of several other MD related classifiers in this list, such as IRS2 [62], PTGS2 [63], DCN [64] and FGF1 [65], further supports the interest of the classifiers as biomarkers of MD development in sacubitril/valsartan good responders.

**4. Analysis of proposed biomarkers with GUILDify**

In the previous section, we proposed 30 proteins that could potentially help to identify HF patients at risk of developing MD. To corroborate these biomarkers, we tested how many of them are found using a different approach also based on the use of functional networks. For this purpose, we used GUILDify v2.0 [66], a web server that extends the information of disease-gene associations through the protein-protein interactions network. GUILDify scores proteins according to their proximity with the genes associated with a disease (seeds). Using this web server, we identify a list of top-scoring proteins that are critical on transmitting the perturbation of disease genes through the network. The network used by GUILDify is completely independent from the HPN used in the TPMS, becoming an ideal, independent context to test the potential biomarkers.

Thus, we used GUILDify to indicate which of the potential biomarkers identified by TPMS may have a relevant role in the molecular mechanism of the drug. We ran GUILDify using the two targets of sacubitril/valsartan (NEP, AT1R) as seeds, and selected the top 2% scored nodes (defined as the “top-drug” set). We did the same with the phenotypes of HF and MD, using as seeds the 124 effectors of HF and 163 effectors of MD from the BED database. We merged the top scored sets of HF, MD and top-drug (“top-drug ∪ top-HF ∪ top-MD”) and studied the overlap with the set of differential best-classifier proteins associated with MD and HF. **Supplementary Table 8** shows the result of this analysis, with a significant representation of best-classifier proteins in most of the sets, especially on MD best-classifier proteins. **Supplementary Table 9** shows the list of 13 proteins involved in this overlap. We have also checked the overlap with the 30 biomarkers proposed in the previous section, of which 10 are found in the merged set “top-drug ∪ top-HF ∪ top-MD” and are consequently significant (see **Supplementary Tables 10 and 11**).

Some of these candidates can be functionally linked to both diseases and the drug under study. For example, among these 10 classifiers, AGER has been implicated in both HF [67], through extracellular matrix remodeling, and MD development [68], through inflammation, oxidative stress, and basal laminar deposit formation between retinal pigment epithelium cells and the basal membrane; furthermore, this receptor is known to be modulated by AT1R [69], valsartan target. Similarly, FGF1 has been proposed to improve cardiac function after HF [70], as well as to promote choroid neovascularization leading to MD [54]. Moreover, FGF1 is regulated by angiotensin II through ATGR2 [71], another protein suggested as classifier in the current analysis that is known to mediate some of the effects of AT1R antagonists, such as valsartan [48,49]. Another candidate, NRG1, has been linked to myocardial regeneration after HF [72] and is known to lessen the development of neurodegenerative diseases such as Alzheimer’s disease [73], which shares similar pathological features with MD [74]. NRG1 is also linked to the expression of neprilysin [73], sacubitril target. ITGB5 has been identified as risk locus for HF [75] and its modulation has been linked to lipofucsin accumulation in MD [76]. Interestingly, ATGR1 inhibitors have been reported to modulate ITGB5 expression in animal models [77]. Finally, IL1A has been proposed as an essential mediator of HF pathogenesis [78,79] through inflammation modulations, and serum levels of this protein have been found increased in MD patients [80]. In addition, as described in previous sections, classifiers FGB, SERPINE1, and SERPING1 have been linked to MD [57–59] and are also known to play a role in HF development [81–84]. According to these findings, the 10 potential biomarkers proposed by TPMS and identified with GUILDify might be prioritized when studying good responder HF patients at risk of MD development.

Supplementary Figures

1. **(a)** **(b)**
2.
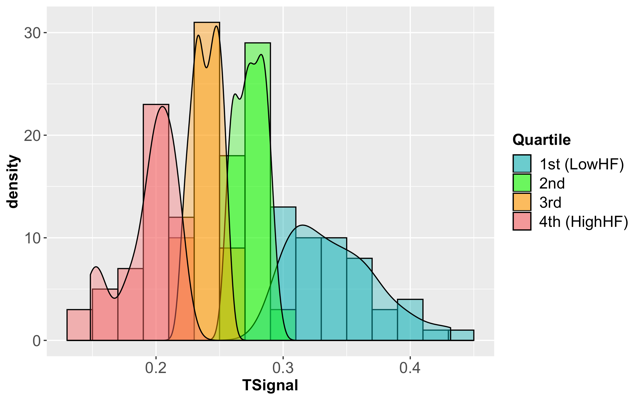

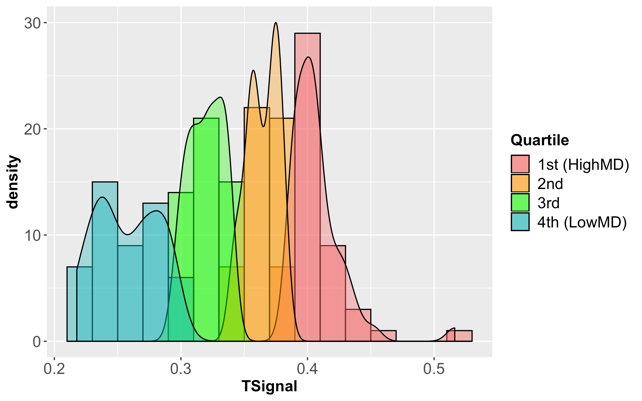

3. **Supplementary Fig 1:** Histogram of the number of models belonging to High- and Low- (HF in **(a)** and MD in **(b)**) in a range of TSignal values. The models are divided in four quartiles, the 1^st^ and 4^th^ corresponding to the Low- and High- groups for HF and vice versa for MD.
4.
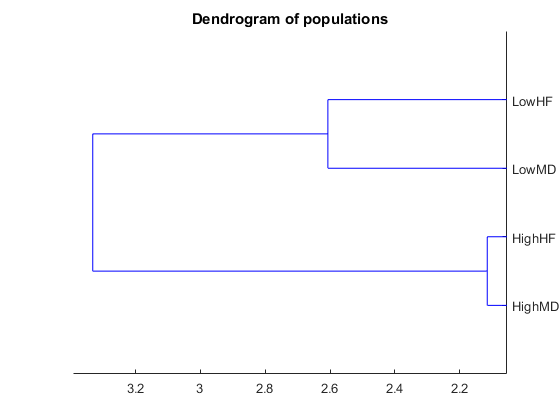

5. **Supplementary Fig 2**: Dendrogram plot of the pairwise modified Hausdorff distance (MHD) between the four groups of mechanisms of action (MoAs): LowHF, HighHF, LowMD, HighMD.

**(a)** **(b)**


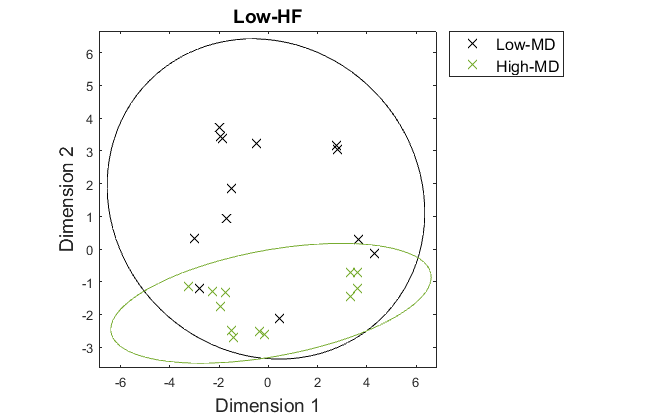

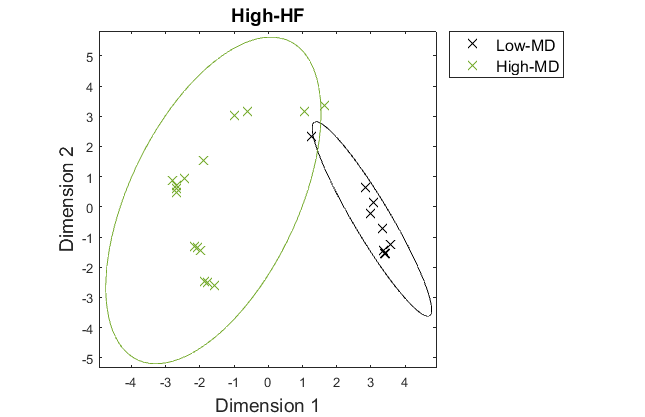


**Supplementary Fig 3:** **Multidimensional scaling plot of the distances between the Mechanisms of Action of Low/High-Macular Degeneration (MD) and Heart Failure (HF)**. In **(a)** we find the models of both MD groups and Low-HF, whereas in **(b)** the models are for both MD groups and High-HF.

**(a)** **(b)**


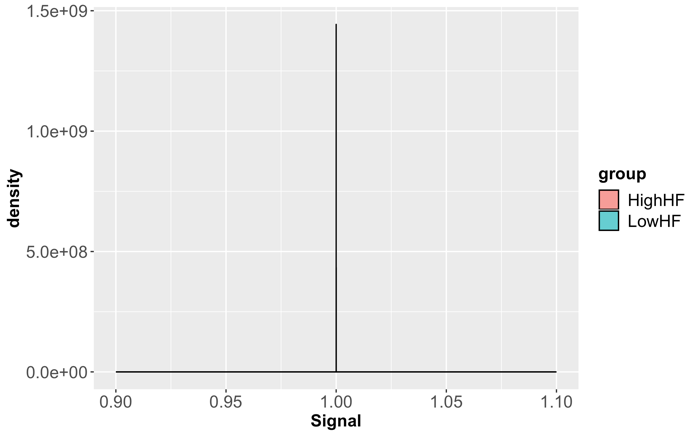

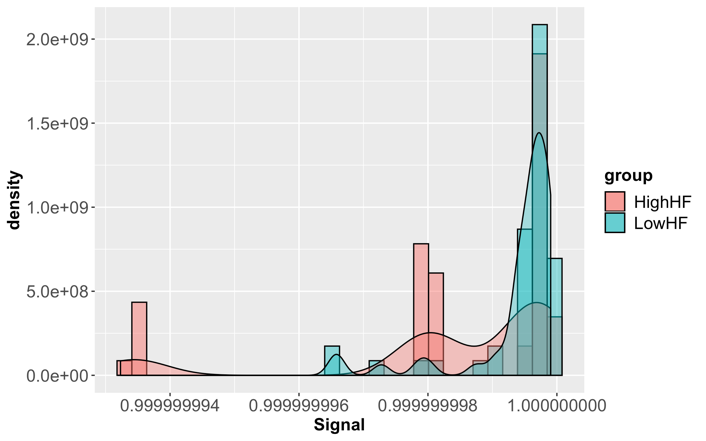


**Supplementary Fig 4:** Histogram of the signal of protein P29353 (non-differential Best-Classifier Protein) in the Mechanisms of Action belonging to High-HF and Low-HF. In **(a)** a general vision of the signal, in **(b)** a detailed vision of the signal.
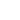


**
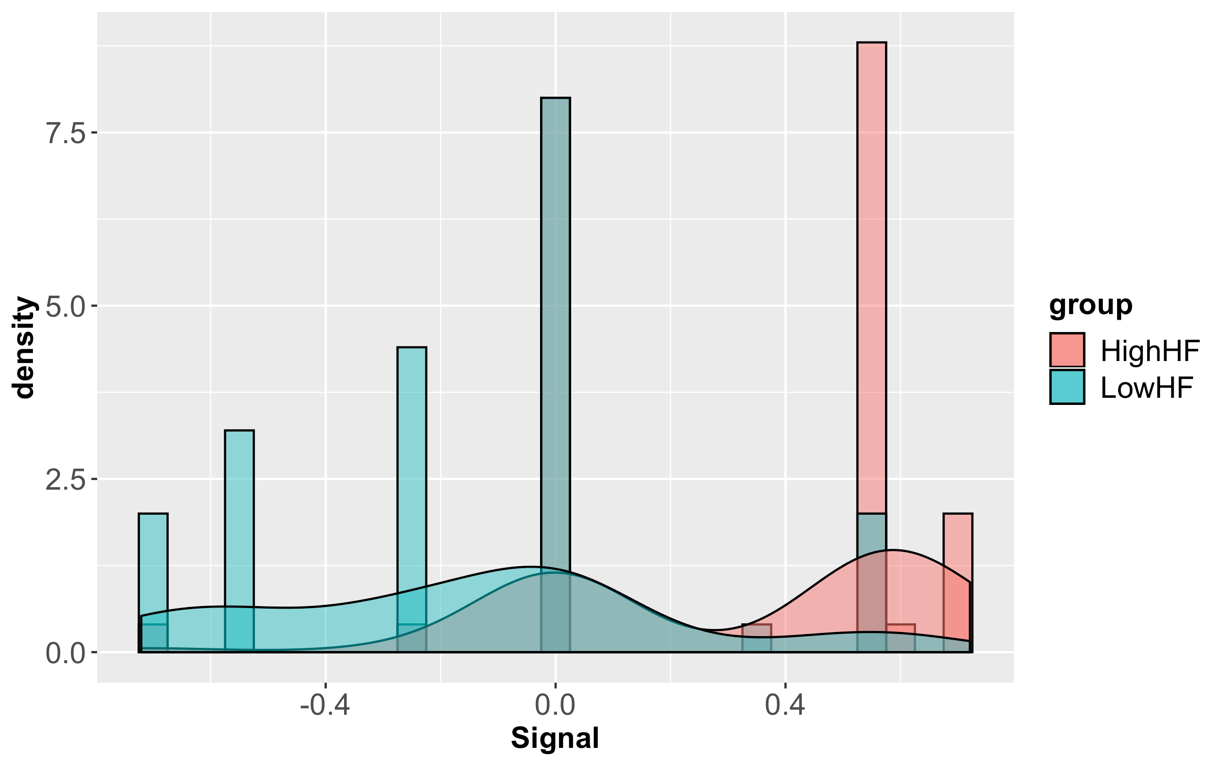
**

**Supplementary Fig 5:** Histogram of the signal of protein P40763 (differential non-Best-Classifier Protein) in the Mechanisms of Action belonging to High-HF and Low-HF.
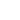


Supplementary Tables

**Supplementary Table 1**: Differential best-classifier proteins with opposite signal in Low-HF (LHF) and High-HF (HHF). “+” stands for active, while “-” stands for inactive. Highlighted cells correspond to proteins that are part of the Top-HF ∪ Top-MD ∪ Top-Drug set, the top-scoring proteins according to GUILDify

|  | **Uniprot ID** | **Gene symbol** | **Gene name** | $\left\langle\boldsymbol{LHF} \right\rangle$ | $\left\langle\boldsymbol{HHF} \right\rangle$ | $\sqrt{\left\vert\boldsymbol{LMDxHMD} \right\vert}$ | **Adjusted P-value** |
| --- | --- | --- | --- | --- | --- | --- | --- |
| **LHF+ HHF-** | Q96F07 | CYFIP2 | Cytoplasmic FMR1-interacting protein 2 | 0.110 | -0.278 | 0.175 | 6.388E-07 |
|  | P55160 | NCKAP1L | Nck-associated protein 1-like {ECO:0000305} | 0.110 | -0.278 | 0.175 | 6.388E-07 |
|  | Q7L576 | CYFIP1 | Cytoplasmic FMR1-interacting protein 1 | 0.110 | -0.278 | 0.175 | 6.388E-07 |
|  | Q9NYB9 | ABI2 | Abl interactor 2 | 0.110 | -0.278 | 0.175 | 6.388E-07 |
|  | Q9Y2A7 | NCKAP1 | Nck-associated protein 1 | 0.110 | -0.278 | 0.175 | 6.388E-07 |
|  | P50052 | AGTR2 | Type-2 angiotensin II receptor | 0.205 | -0.013 | 0.051 | 1.852E-05 |
| **LHF- HHF+** | **P28482** | **MAPK1** | **Mitogen-activated protein kinase 1** | -0.710 | 0.479 | 0.584 | 1.854E-14 |
|  | **P27361** | **MAPK3** | **Mitogen-activated protein kinase 3** | -0.313 | 0.962 | 0.549 | 6.366E-15 |
|  | P47900 | P2RY1 | P2Y purinoceptor 1 | -0.322 | 0.605 | 0.441 | 9.855E-10 |
|  | Q92558 | WASF1 | Wiskott-Aldrich syndrome protein family member 1 | -0.580 | 0.309 | 0.424 | 8.494E-13 |
|  | Q9Y6W5 | WASF2 | Wiskott-Aldrich syndrome protein family member 2 | -0.580 | 0.309 | 0.424 | 8.494E-13 |
|  | O00401 | WASL | Neural Wiskott-Aldrich syndrome protein | -0.580 | 0.309 | 0.424 | 8.494E-13 |
|  | **P02751** | **FN1** | **Fibronectin** | -0.476 | 0.224 | 0.327 | 2.152E-09 |
|  | O60229 | KALRN | Kalirin {ECO:0000250\|UniProtKB:P97924} | -0.529 | 0.185 | 0.313 | 3.239E-05 |
|  | Q8TEW0 | PARD3 | Partitioning defective 3 homolog | -0.279 | 0.255 | 0.267 | 3.577E-09 |
|  | P41743 | PRKCI | Protein kinase C iota type | -0.279 | 0.255 | 0.267 | 3.577E-09 |
|  | Q13576 | IQGAP2 | Ras GTPase-activating-like protein IQGAP2 | -0.279 | 0.255 | 0.267 | 3.577E-09 |
|  | O75914 | PAK3 | Serine/threonine-protein kinase PAK 3 | -0.279 | 0.255 | 0.267 | 3.577E-09 |
|  | Q9P286 | PAK5 | Serine/threonine-protein kinase PAK 5 {ECO:0000305} | -0.279 | 0.255 | 0.267 | 3.577E-09 |
|  | O96013 | PAK4 | Serine/threonine-protein kinase PAK 4 | -0.279 | 0.255 | 0.267 | 3.577E-09 |
|  | Q86VI3 | IQGAP3 | Ras GTPase-activating-like protein IQGAP3 | -0.279 | 0.255 | 0.267 | 3.577E-09 |
|  | Q9NPB6 | PARD6A | Partitioning defective 6 homolog alpha | -0.279 | 0.255 | 0.267 | 3.577E-09 |
|  | Q16584 | MAP3K11 | Mitogen-activated protein kinase kinase kinase 11 | -0.279 | 0.255 | 0.267 | 3.577E-09 |
|  | Q9NQU5 | BUB1B-PAK6; PAK6 | Serine/threonine-protein kinase PAK 6 | -0.279 | 0.255 | 0.267 | 3.577E-09 |
|  | Q9BYG5 | PARD6B | Partitioning defective 6 homolog beta | -0.279 | 0.255 | 0.267 | 3.577E-09 |
|  | Q9BYG4 | PARD6G | Partitioning defective 6 homolog gamma | -0.279 | 0.255 | 0.267 | 3.577E-09 |
|  | P84095 | RHOG | Rho-related GTP-binding protein RhoG | -0.449 | 0.156 | 0.265 | 1.935E-09 |
|  | Q96PN6 | ADCY10 | Adenylate cyclase type 10 | -0.488 | 0.144 | 0.265 | 2.421E-09 |
|  | Q96JJ3 | ELMO2 | Engulfment and cell motility protein 2 | -0.396 | 0.137 | 0.233 | 1.935E-09 |
|  | Q15759 | MAPK11 | Mitogen-activated protein kinase 11 | -0.883 | 0.056 | 0.223 | 6.366E-15 |
|  | O15264 | MAPK13 | Mitogen-activated protein kinase 13 | -0.884 | 0.045 | 0.198 | 6.366E-15 |
|  | P53778 | MAPK12 | Mitogen-activated protein kinase 12 | -0.884 | 0.045 | 0.198 | 6.366E-15 |
|  | P54764 | EPHA4 | Ephrin type-A receptor 4 | -0.139 | 0.233 | 0.180 | 6.388E-07 |
|  | Q99755 | PIP5K1A | Phosphatidylinositol 4-phosphate 5-kinase type-1 alpha | -0.110 | 0.278 | 0.175 | 6.388E-07 |
|  | Q9UQB8 | BAIAP2 | Brain-specific angiogenesis inhibitor 1-associated protein 2 | -0.110 | 0.278 | 0.175 | 6.388E-07 |
|  | O14986 | PIP5K1B | Phosphatidylinositol 4-phosphate 5-kinase type-1 beta | -0.110 | 0.278 | 0.175 | 6.388E-07 |
|  | Q9Y5S8 | NOX1 | NADPH oxidase 1 | -0.110 | 0.278 | 0.175 | 6.388E-07 |
|  | P46734 | MAP2K3 | Dual specificity mitogen-activated protein kinase kinase 3 | -0.110 | 0.278 | 0.175 | 6.388E-07 |
|  | Q15080 | NCF4 | Neutrophil cytosol factor 4 | -0.110 | 0.278 | 0.175 | 6.388E-07 |
|  | Q9HBY0 | NOX3 | NADPH oxidase 3 | -0.110 | 0.278 | 0.175 | 6.388E-07 |
|  | Q9UPY6 | WASF3 | Wiskott-Aldrich syndrome protein family member 3 | -0.110 | 0.278 | 0.175 | 6.388E-07 |
|  | P52564 | MAP2K6 | Dual specificity mitogen-activated protein kinase kinase 6 | -0.110 | 0.278 | 0.175 | 6.388E-07 |
|  | O14733 | MAP2K7 | Dual specificity mitogen-activated protein kinase kinase 7 | -0.110 | 0.278 | 0.175 | 6.388E-07 |
|  | Q9Y4K3 | TRAF6 | TNF receptor-associated factor 6 | -0.097 | 0.285 | 0.166 | 1.917E-06 |
|  | P19878 | NCF2 | Neutrophil cytosol factor 2 | -0.042 | 0.278 | 0.108 | 3.050E-05 |

**Supplementary Table 2**: Top 10 gene Ontology functions enriched from best-classifier proteins with opposite signal in Heart Failure (HF) MoAs. Functional enrichment analysis from FuncAssociate.

|  | **Low-HF active / High-HF inactive** | | | **Low-HF inactive / High-HF active** | | | **Overlapped functions** | | |
| --- | --- | --- | --- | --- | --- | --- | --- | --- | --- |
|  | **GO name** | **LOD** | **P-val.** | **GO name** | **LOD** | **P-val.** | **GO name** | **LOD** | **P-val.** |
| **1** | SCAR complex | 3.89 | <0.00050 | 1-phosphatidylinositol-3-phosphate 5-kinase activity | 3.41 | 0.01700 | Rac protein signal transduction | 2.54 | <0.00050 |
| **2** | positive regulation of Arp2/3 complex-mediated actin nucleation | 3.64 | <0.00050 | 1-phosphatidylinositol-5-kinase activity | 3.41 | 0.01700 | vascular endothelial growth factor receptor signaling pathway | 2.31 | <0.00050 |
| **3** | positive regulation of neurotrophin TRK receptor signaling pathway | 3.49 | 0.00150 | phosphatidylinositol-3,4-bisphosphate 5-kinase activity | 2.94 | 0.04000 | immune response-regulating cell surface receptor signaling pathway involved in phagocytosis | 1.95 | <0.00050 |
| **4** | regulation of neurotrophin TRK receptor signaling pathway | 3.36 | <0.00050 | proteolysis in other organism | 2.73 | 0.00250 | Fc-gamma receptor signaling pathway involved in phagocytosis | 1.95 | <0.00050 |
| **5** | positive regulation of actin nucleation | 3.27 | <0.00050 | MAP kinase kinase activity | 2.60 | <0.00050 | Fc receptor mediated stimulatory signaling pathway | 1.95 | <0.00050 |
| **6** | regulation of Arp2/3 complex-mediated actin nucleation | 3.23 | <0.00050 | NADPH oxidase complex | 2.58 | <0.00050 | Fc-gamma receptor signaling pathway | 1.94 | <0.00050 |
| **7** | dendrite extension | 3.16 | 0.00350 | DNA damage induced protein phosphorylation | 2.53 | 0.00450 | Fc receptor signaling pathway | 1.74 | <0.00050 |
| **8** | regulation of actin nucleation | 2.96 | <0.00050 | MAP kinase activity | 2.52 | <0.00050 | Ras protein signal transduction | 1.79 | <0.00050 |
| **9** | filopodium tip | 2.84 | 0.01200 | superoxide-generating NADPH oxidase activity | 2.34 | 0.00600 | regulation of actin cytoskeleton organization | 1.62 | 0.00072 |
| **10** | developmental cell growth | 2.42 | 0.00150 | superoxide anion generation | 2.25 | 0.00850 | lamellipodium | 1.71 | 0.00086 |

**Supplementary Table 3**: Differential best-classifier proteins with opposite signal in Low-MD (LMD) and High-MD (HMD). “+” stands for active, while “-” stands for inactive. Highlighted cells correspond to proteins that are part of the Top-HF ∪ Top-MD ∪ Top-Drug set, the top-scoring proteins according to GUILDify

|  | **Uniprot ID** | **Gene symbol** | **Gene name** | $\left\langle\boldsymbol{LMD} \right\rangle$ | $\left\langle\boldsymbol{HMD} \right\rangle$ | $\sqrt{\left\vert\boldsymbol{LMDxHMD} \right\vert}$ | **Adjusted P-value** |
| --- | --- | --- | --- | --- | --- | --- | --- |
| **LMD+ HMD-** | Q9Y4H2 | IRS2 | Insulin receptor substrate 2 | 0.583 | -0.414 | 0.491 | 1.297E-13 |
|  | O43639 | NCK2 | Cytoplasmic protein NCK2 | 0.623 | -0.355 | 0.471 | 5.744E-11 |
|  | Q13153 | PAK1 | Serine/threonine-protein kinase PAK 1 {ECO:0000303\|PubMed:8805275} | 0.233 | -0.817 | 0.437 | 2.266E-12 |
|  | P30530 | AXL | Tyrosine-protein kinase receptor UFO | 0.476 | -0.362 | 0.415 | 5.509E-16 |
|  | P42081 | CD86 | T-lymphocyte activation antigen CD86 | 0.428 | -0.356 | 0.391 | 2.073E-08 |
|  | P18825 | ADRA2C | Alpha-2C adrenergic receptor | 0.226 | -0.568 | 0.358 | 2.079E-10 |
|  | Q13177 | PAK2 | Serine/threonine-protein kinase PAK 2 | 0.249 | -0.439 | 0.330 | 3.753E-09 |
|  | P54762 | EPHB1 | Ephrin type-B receptor 1 | 0.144 | -0.685 | 0.314 | 2.916E-14 |
|  | P15498 | VAV1 | Proto-oncogene vav | 0.392 | -0.192 | 0.274 | 8.020E-05 |
|  | P06241 | FYN | Tyrosine-protein kinase Fyn | 0.589 | -0.127 | 0.274 | 7.322E-15 |
|  | **O75787** | **ATP6AP2** | **V-ATPase M8.9 subunit** | 0.407 | -0.160 | 0.255 | 2.741E-08 |
|  | **P01583** | **IL1A** | **Interleukin-1 alpha** | 0.125 | -0.396 | 0.222 | 2.087E-12 |
|  | **P06748** | **NPM1** | **Nucleophosmin** | 0.374 | -0.116 | 0.208 | 2.266E-12 |
|  | **Q02297** | **NRG1** | **Pro-neuregulin-1, membrane-bound isoform** | 0.670 | -0.064 | 0.207 | 5.208E-14 |
|  | P15941 | MUC1 | Mucin-1 subunit alpha | 0.085 | -0.479 | 0.202 | 1.676E-11 |
|  | **P18084** | **ITGB5** | **Integrin beta-5** | 0.498 | -0.079 | 0.199 | 1.214E-15 |
|  | P03372 | ESR1 | Estrogen receptor | 0.096 | -0.294 | 0.169 | 6.103E-08 |
|  | P01138 | NGF | Beta-nerve growth factor | 0.211 | -0.124 | 0.162 | 6.954E-07 |
|  | P43405 | SYK | Tyrosine-protein kinase SYK | 0.075 | -0.310 | 0.152 | 1.618E-07 |
|  | Q08722 | CD47 | Leukocyte surface antigen CD47 | 0.082 | -0.277 | 0.151 | 8.239E-07 |
|  | P54764 | EPHA4 | Ephrin type-A receptor 4 | 0.336 | -0.065 | 0.148 | 4.859E-08 |
|  | Q9BYF1 | ACE2 | Processed angiotensin-converting enzyme 2 | 0.565 | -0.039 | 0.148 | 7.333E-15 |
|  | P10275 | AR | Androgen receptor | 0.438 | -0.045 | 0.141 | 1.014E-11 |
|  | P38398 | BRCA1 | Breast cancer type 1 susceptibility protein | 0.043 | -0.365 | 0.125 | 9.363E-08 |
|  | P35354 | PTGS2 | Prostaglandin G/H synthase 2 | 0.034 | -0.396 | 0.116 | 2.482E-12 |
|  | Q9Y478 | PRKAB1 | 5'-AMP-activated protein kinase subunit beta-1 | 0.374 | -0.034 | 0.113 | 5.744E-11 |
|  | P14770 | GP9 | Platelet glycoprotein IX | 0.034 | -0.306 | 0.102 | 1.190E-08 |
|  | P14138 | EDN3 | Endothelin-3 | 0.023 | -0.239 | 0.074 | 3.509E-06 |
| **LMD- HMD+** | **P02675** | **FGB** | **Fibrinogen beta chain** | -0.778 | 0.654 | 0.713 | 3.040E-14 |
|  | O60674 | JAK2 | Tyrosine-protein kinase JAK2 | -0.811 | 0.279 | 0.476 | 2.749E-16 |
|  | **P04085** | **PDGFA** | **Platelet-derived growth factor subunit A** | -0.359 | 0.622 | 0.473 | 1.263E-07 |
|  | Q05586 | GRIN1 | Glutamate receptor ionotropic, NMDA 1 | -0.381 | 0.565 | 0.464 | 1.049E-15 |
|  | **P05230** | **FGF1** | **Fibroblast growth factor 1** | -0.219 | 0.734 | 0.401 | 1.528E-14 |
|  | Q15768 | EFNB3 | Ephrin-B3 | -0.149 | 0.835 | 0.353 | 8.307E-13 |
|  | Q14451 | GRB7 | Growth factor receptor-bound protein 7 | -0.181 | 0.679 | 0.351 | 5.106E-13 |
|  | P08581 | MET | Hepatocyte growth factor receptor | -0.124 | 0.828 | 0.321 | 2.615E-13 |
|  | Q08289 | CACNB2 | Voltage-dependent L-type calcium channel subunit beta-2 | -0.351 | 0.238 | 0.289 | 5.549E-09 |
|  | P63244 | RACK1 | Receptor of activated protein C kinase 1, N-terminally processed | -0.395 | 0.206 | 0.285 | 4.410E-08 |
|  | Q00987 | MDM2 | E3 ubiquitin-protein ligase Mdm2 | -0.458 | 0.166 | 0.275 | 2.789E-08 |
|  | P32004 | L1CAM | Neural cell adhesion molecule L1 | -0.466 | 0.118 | 0.235 | 2.519E-12 |
|  | P15391 | CD19 | B-lymphocyte antigen CD19 | -0.272 | 0.171 | 0.216 | 4.123E-08 |
|  | P07948 | LYN | Tyrosine-protein kinase Lyn | -0.109 | 0.408 | 0.211 | 3.099E-04 |
|  | O14745 | SLC9A3R1 | Na(+)/H(+) exchange regulatory cofactor NHE-RF1 | -0.172 | 0.224 | 0.196 | 4.627E-07 |
|  | O43559 | FRS3 | Fibroblast growth factor receptor substrate 3 | -0.091 | 0.317 | 0.170 | 3.717E-08 |
|  | P43146 | DCC | Netrin receptor DCC | -0.392 | 0.070 | 0.165 | 5.835E-04 |
|  | P62158 | CALM1 ; CALM2 ; CALM3 | Calmodulin-1 {ECO:0000312\|HGNC:HGNC:1442} | -0.455 | 0.054 | 0.156 | 1.670E-10 |
|  | **P42574** | **CASP3** | **Caspase-3 subunit p12** | -0.034 | 0.656 | 0.149 | 8.050E-08 |
|  | P42684 | ABL2 | Abelson tyrosine-protein kinase 2 | -0.362 | 0.045 | 0.128 | 1.676E-11 |
|  | P17081 | RHOQ | Rho-related GTP-binding protein RhoQ | -0.362 | 0.045 | 0.128 | 1.676E-11 |
|  | Q13905 | RAPGEF1 | Rap guanine nucleotide exchange factor 1 | -0.187 | 0.080 | 0.122 | 2.094E-04 |
|  | **P05155** | **SERPING1** | **Plasma protease C1 inhibitor** | -0.023 | 0.362 | 0.091 | 1.014E-11 |
|  | Q92793 | CREBBP | CREB-binding protein | -0.506 | 0.015 | 0.089 | 4.511E-11 |
|  | P07585 | DCN | Decorin | -0.023 | 0.351 | 0.089 | 2.430E-11 |
|  | P12830 | CDH1 | Cadherin-1 | -0.503 | 0.011 | 0.076 | 1.487E-14 |
|  | Q07157 | TJP1 | Tight junction protein ZO-1 | -0.407 | 0.011 | 0.068 | 2.640E-12 |
|  | Q92990 | GLMN | Glomulin | -0.294 | 0.011 | 0.058 | 2.056E-07 |
|  | P55075 | FGF8 | Fibroblast growth factor 8 | -0.011 | 0.238 | 0.052 | 1.884E-05 |

**Supplementary Table 4**: Top 10 gene Ontology functions enriched from best-classifier proteins with opposite signal in Macular Degeneration (MD) MoAs. Functional enrichment analysis from FuncAssociate.

|  | **Low-MD active / High-MD inactive** | | | | **Low-MD inactive / High-MD active** | | | | **Overlapped functions** | | |
| --- | --- | --- | --- | --- | --- | --- | --- | --- | --- | --- | --- |
|  | **GO name** | **LOD** | **P-val.** | **GO name** | | **LOD** | **P-val.** | **GO name** | | **LOD** | **P-val.** |
| **1** | dendritic spine development | 2.41 | 0.00150 | dorsal/ventral axon guidance | | 3.07 | 0.01950 | phosphatidylinositol-4,5-bisphosphate 3-kinase activity | | 1.89 | <0.00050 |
| **2** | positive regulation of vascular endothelial growth factor production | 2.04 | 0.03000 | fibroblast growth factor receptor binding | | 2.06 | 0.02000 | phosphatidylinositol bisphosphate kinase activity | | 1.87 | <0.00050 |
| **3** | regulation of intracellular estrogen receptor signaling pathway | 2.00 | 0.00150 | platelet-derived growth factor receptor signaling pathway | | 1.95 | 0.03350 | phosphatidylinositol 3-kinase activity | | 1.84 | <0.00050 |
| **4** | regulation of systemic arterial blood pressure | 1.97 | 0.03300 | non-membrane spanning protein tyrosine kinase activity | | 1.88 | 0.04000 | phosphatidylinositol phosphorylation | | 1.72 | <0.00050 |
| **5** | regulation of vascular endothelial growth factor production | 1.96 | 0.04450 | growth factor receptor binding | | 1.85 | <0.00050 | single-organism cellular process | | 1.53 | <0.00050 |
| **6** | peptide hormone processing | 1.96 | 0.04450 | regulation of blood coagulation | | 1.68 | 0.01050 | lipid phosphorylation | | 1.67 | <0.00050 |
| **7** | phosphotyrosine binding | 1.91 | 0.04900 | regulation of hemostasis | | 1.68 | 0.01050 | positive regulation of protein kinase B signaling | | 1.60 | <0.00050 |
| **8** | neutrophil chemotaxis | 1.90 | 0.05000 | regulation of coagulation | | 1.66 | 0.01050 | biological regulation | | 1.42 | <0.00050 |
| **9** | regulation of vasoconstriction | 1.89 | 0.00150 | regulation of phosphatidylinositol 3-kinase signaling | | 1.58 | 0.02350 | protein binding | | 1.41 | <0.00050 |
| **10** | vascular endothelial growth factor receptor signaling pathway | 1.83 | <0.00050 | response to toxic substance | | 1.53 | 0.00350 | regulation of response to stimulus | | 1.24 | <0.00050 |

**Supplementary Table 5**: Modified Hausdorff distance between the 4 groups of MoAs defined.

|  | LowMD | HighMD | HighHF | LowHF |
| --- | --- | --- | --- | --- |
| LowMD | 0 | 4.00226983 | 2.7537393 | 2.6068664 |
| HighMD | 4.00226983 | 0 | 2.1150102 | 2.55445687 |
| HighHF | 2.7537393 | 2.1150102 | 0 | 4.01919608 |
| LowHF | 2.6068664 | 2.55445687 | 4.01919608 | 0 |

**Supplementary Table 6**: Mean Euclidean distance between each one of the points of every group of MoAs and its centre.

|  | Mean distance from center |
| --- | --- |
| LowMD | 3.137818031 |
| HighMD | 3.171767895 |
| HighHF | 3.298746704 |
| LowHF | 3.523965485 |

**Supplementary Table 7**: Number of common MoAs between the 4 groups of MoAs defined.

|  | LowMD | HighMD | HighHF | LowHF |
| --- | --- | --- | --- | --- |
| LowMD | 50 | 0 | 9 | 13 |
| HighMD | 0 | 50 | 17 | 12 |
| HighHF | 9 | 17 | 50 | 0 |
| LowHF | 13 | 12 | 0 | 50 |

**Supplementary Table 8**: Intersection of several set of proteins defined with GUILDify with the best-classifier proteins (BCP) obtained from the TPMS analysis. The p-values are calculated using a Fisher’s exact test. The p-values above 0.05 are remarked in red.

| **Sets of proteins** | **# LHF+ HHF-** | **P-value** | **# LHF- HHF+** | **P-value** | **# LMD+ HMD-** | **P-value** | **# LMD- HMD+** | **P-value** |
| --- | --- | --- | --- | --- | --- | --- | --- | --- |
| Drug seeds | 0 | 1.00E+00 | 0 | 1.00E+00 | 0 | 1.00E+00 | 0 | 1.00E+00 |
| Top-Drug | 0 | 1.00E+00 | 0 | 1.00E+00 | 2 | 1.11E-01 | 0 | 1.00E+00 |
| HF seeds | 0 | 1.00E+00 | 3 | 6.32E-03 | 1 | 2.32E-01 | 1 | 2.39E-01 |
| Top-HF | 0 | 1.00E+00 | 3 | 4.34E-02 | 2 | 1.02E-01 | 1 | 4.35E-01 |
| MD seeds | 0 | 1.00E+00 | 1 | 4.02E-01 | 2 | 4.81E-02 | 5 | 2.76E-05 |
| Top-MD | 0 | 1.00E+00 | 1 | 5.60E-01 | 3 | 1.81E-02 | 5 | 2.51E-04 |
| **Top-HF∪Top-MD∪Top-Drug** | **0** | 1.00E+00 | **3** | 3.70E-01 | **5** | 1.53E-02 | **5** | 1.77E-02 |

**Supplementary Table 9**: Best-classifier proteins found in the Top-HF ∪ Top-MD ∪ Top-Drug set.

|  | **Uniprot ID** | **Gene symbol** | **Gene name** |
| --- | --- | --- | --- |
| **LHF-**  **HHF+** | P28482 | MAPK1 | Mitogen-activated protein kinase 1 |
|  | P27361 | MAPK3 | Mitogen-activated protein kinase 3 |
|  | P02751 | FN1 | Fibronectin |
| **LMD+**  **HMD-** | P18084 | ITGB5 | Integrin beta-5 |
|  | O75787 | ATP6AP2 | V-ATPase M8.9 subunit |
|  | Q02297 | NRG1 | Pro-neuregulin-1, membrane-bound isoform |
|  | P06748 | NPM1 | Nucleophosmin |
|  | P01583 | IL1A | Interleukin-1 alpha |
| **LMD- HMD+** | P04085 | PDGFA | Platelet-derived growth factor subunit A |
|  | P02675 | FGB | Fibrinogen beta chain |
|  | P05155 | SERPING1 | Plasma protease C1 inhibitor |
|  | P05230 | FGF1 | Fibroblast growth factor 1 |
|  | P42574 | CASP3 | Caspase-3 subunit p12 |

**Supplementary Table 10**: Intersection of several set of proteins defined with GUILDify with the biomarkers obtained from the TPMS analysis. The p-values are calculated using a Fisher’s exact test. The p-values above 0.05 are remarked in red.

| **Sets of proteins** | **LHF** ∩ **LMD+ HMD-** | **P-value** | **LHF** ∩ **LMD- HMD+** | **P-value** |
| --- | --- | --- | --- | --- |
| Drug seeds | 0 | 1.00E+00 | 0 | 1.00E+00 |
| Top-Drug | 1 | 2.90E-01 | 0 | 1.00E+00 |
| HF seeds | 1 | 1.45E-01 | 1 | 1.28E-01 |
| Top-HF | **2** | 4.03E-02 | 1 | 2.48E-01 |
| MD seeds | **2** | 1.80E-02 | **4** | 2.54E-05 |
| Top-MD | **4** | 2.75E-04 | **4** | 1.56E-04 |
| Top-HF∪Top-MD∪Top-Drug | **5** | 1.37E-03 | **5** | 6.89E-04 |

**Supplementary Table 11**: Biomarkers from the TPMS analysis found in the Top-HF ∪ Top-MD ∪ Top-Drug set.

|  | **Uniprot ID** | **Gene symbol** | **Gene name** |
| --- | --- | --- | --- |
| **LHF** ∩ **LMD+ HMD-** | Q02297 | NRG1 | Pro-neuregulin-1, membrane-bound isoform |
|  | P06748 | NPM1 | Nucleophosmin |
|  | P01583 | IL1A | Interleukin-1 alpha |
|  | P61981 | YWHAG | 14-3-3 protein gamma, N-terminally processed |
|  | P18084 | ITGB5 | Integrin beta-5 |
| **LHF** ∩ **LMD- HMD+** | P05121 | SERPINE1 | Plasminogen activator inhibitor 1 |
|  | P02675 | FGB | Fibrinogen beta chain |
|  | P05230 | FGF1 | Fibroblast growth factor 1 |
|  | Q15109 | AGER | Advanced glycosylation end product-specific receptor |
|  | P05155 | SERPING1 | Plasma protease C1 inhibitor |

**Supplementary Table 12**: Pathophysiological processes present in Heart Failure characterization used in the study.

| **Pathophysiological processes** | **# proteins** |
| --- | --- |
| 1-      Cardiomyocyte cell death (including apoptosis and necrosis) | 46 |
| 2-      Left ventricle extracellular matrix remodelling | 37 |
| 3-      Impaired myocyte contractility | 35 |
| 4-      Hypertrophy | 33 |

**Supplementary Table 13**: Pathophysiological processes present in Macular Degeneration characterization used in the study.

| **Pathophysiological processes** | **# proteins** |
| --- | --- |
| 1- Light and oxidative stress: lipid oxidation, lipofuscin, advanced glycation end products (AGEs) | 46 |
| 2- Debris accumulation: Drusen and protein aggregation | 37 |
| 3- Disturbance of lysosomal clearance | 35 |
| 4- Autophagy dysregulation | 33 |
| 5- Immunological processes: chronic inflammation | 46 |
| 6- Mitochondrial defects | 37 |
| 7- Extracellular matrix remodelling and Bruch's membrane thickening | 35 |
| 8- Lipoprotein/lipid metabolism | 33 |
| 9- Retinal cell death | 35 |
| 10- Choroidal neovascularization (wet Age-Related MD) | 33 |

References

1. Anaxomics Biotech SL. Biological Effectors Database [Internet]. 2018. Available: http://www.anaxomics.com/biological-effectors-database.php

2. Pujol A, Mosca R, Farrés J, Aloy P. Unveiling the role of network and systems biology in drug discovery. Trends Pharmacol Sci. 2010;31: 115–123. doi:10.1016/j.tips.2009.11.006

3. Iborra-Egea O, Gálvez-Montón C, Roura S, Perea-Gil I, Prat-Vidal C, Soler-Botija C, et al. Mechanisms of action of sacubitril/valsartan on cardiac remodeling: a systems biology approach. npj Syst Biol Appl. 2017;3: 1–8. doi:10.1038/s41540-017-0013-4

4. European Medicines Agency. Entresto: EPAR - Public assessment report [Internet]. London; 2015. Available: https://www.ema.europa.eu/en/documents/assessment-report/entresto-epar-public-assessment-report_en.pdf

5. Leger F, Fernagut PO, Canron MH, Léoni S, Vital C, Tison F, et al. Protein aggregation in the aging retina. J Neuropathol Exp Neurol. 2011;70: 63–68. doi:10.1097/NEN.0b013e31820376cc

6. Hyttinen JMT, Amadio M, Viiri J, Pascale A, Salminen A, Kaarniranta K. Clearance of misfolded and aggregated proteins by aggrephagy and implications for aggregation diseases. Ageing Research Reviews. 2014. pp. 16–28. doi:10.1016/j.arr.2014.07.002

7. Chiras D, Kitsos G, Petersen MB, Skalidakis I, Kroupis C. Oxidative stress in dry age-related macular degeneration and exfoliation syndrome. Critical Reviews in Clinical Laboratory Sciences. 2015. pp. 12–27. doi:10.3109/10408363.2014.968703

8. Nowak JZ. AMD-the retinal disease with an unprecised etiopathogenesis: In search of effective therapeutics. Acta Pol Pharm - Drug Res. 2014;71: 900–16.

9. Anaxomics Biotech SL. TPMS technology [Internet]. 2018. Available: http://www.anaxomics.com/tpms.php

10. Herrando-Grabulosa M, Mulet R, Pujol A, Mas JM, Navarro X, Aloy P, et al. Novel Neuroprotective Multicomponent Therapy for Amyotrophic Lateral Sclerosis Designed by Networked Systems. PLoS One. 2016;11: e0147626. doi:10.1371/journal.pone.0147626

11. Gómez-Serrano M, Camafeita E, García-Santos E, López JA, Rubio MA, Sánchez-Pernaute A, et al. Proteome-wide alterations on adipose tissue from obese patients as age-, diabetes- and gender-specific hallmarks. Sci Rep. 2016;6: 1–15. doi:10.1038/srep25756

12. Perera S, Artigas L, Mulet R, Mas JM, Sardón T. Systems biology applied to non-alcoholic fatty liver disease (NAFLD): treatment selection based on the mechanism of action of nutraceuticals. Nutrafoods. 2014;13: 61–68. doi:10.1007/s13749-014-0022-5

13. Romeo-Guitart D, Forés J, Herrando-Grabulosa M, Valls R, Leiva-Rodríguez T, Galea E, et al. Neuroprotective Drug for Nerve Trauma Revealed Using Artificial Intelligence. Sci Rep. 2018;8: 1879. doi:10.1038/s41598-018-19767-3

14. Wishart DS, Feunang YD, Guo AC, Lo EJ, Marcu A, Grant JR, et al. DrugBank 5.0: A major update to the DrugBank database for 2018. Nucleic Acids Res. 2018;46: D1074–D1082. doi:10.1093/nar/gkx1037

15. Kim S, Thiessen PA, Bolton EE, Chen J, Fu G, Gindulyte A, et al. PubChem substance and compound databases. Nucleic Acids Res. 2016;44: D1202–D1213. doi:10.1093/nar/gkv951

16. Szklarczyk D, Santos A, Von Mering C, Jensen LJ, Bork P, Kuhn M. STITCH 5: Augmenting protein-chemical interaction networks with tissue and affinity data. Nucleic Acids Res. 2016;44: D380–D384. doi:10.1093/nar/gkv1277

17. Hecker N, Ahmed J, von Eichborn J, Dunkel M, Macha K, Eckert A, et al. SuperTarget goes quantitative: update on drug-target interactions. Nucleic Acids Res. 2011;40: D1113–D1117. doi:10.1093/nar/gkr912

18. Kanehisa M, Furumichi M, Tanabe M, Sato Y, Morishima K. KEGG: New perspectives on genomes, pathways, diseases and drugs. Nucleic Acids Res. 2017;45: D353–D361. doi:10.1093/nar/gkw1092

19. Chatr-Aryamontri A, Oughtred R, Boucher L, Rust J, Chang C, Kolas NK, et al. The BioGRID interaction database: 2017 update. Nucleic Acids Res. 2017;45: D369–D379. doi:10.1093/nar/gkw1102

20. Orchard S, Ammari M, Aranda B, Breuza L, Briganti L, Broackes-Carter F, et al. The MIntAct project - IntAct as a common curation platform for 11 molecular interaction databases. Nucleic Acids Res. 2014;42: 358–363. doi:10.1093/nar/gkt1115

21. Fabregat A, Jupe S, Matthews L, Sidiropoulos K, Gillespie M, Garapati P, et al. The Reactome Pathway Knowledgebase. Nucleic Acids Res. 2018;46: D649–D655. doi:10.1093/nar/gkx1132

22. Han H, Cho JW, Lee S, Yun A, Kim H, Bae D, et al. TRRUST v2: An expanded reference database of human and mouse transcriptional regulatory interactions. Nucleic Acids Res. 2018;46: D380–D386. doi:10.1093/nar/gkx1013

23. Keshava Prasad TS, Goel R, Kandasamy K, Keerthikumar S, Kumar S, Mathivanan S, et al. Human Protein Reference Database - 2009 update. Nucleic Acids Res. 2009;37: D767–D772. doi:10.1093/nar/gkn892

24. Salwinski L, Miller CS, Smith AJ, Pettit FK, Bowie JU, Eisenberg D. The Database of Interacting Proteins: 2004 update. Nucleic Acids Res. 2004;32: 449D – 451. doi:10.1093/nar/gkh086

25. Kuhn M, Letunic I, Jensen LJ, Bork P. The SIDER database of drugs and side effects. Nucleic Acids Res. 2016;44: D1075–D1079. doi:10.1093/nar/gkv1075

26. Liu Y, Morley M, Brandimarto J, Hannenhalli S, Hu Y, Ashley EA, et al. RNA-Seq identifies novel myocardial gene expression signatures of heart failure. Genomics. 2015;105: 83–9. doi:10.1016/j.ygeno.2014.12.002

27. Collet P, Rennard J-P. Stochastic Optimization Algorithms. Intell Inf Technol. 2011; 1121–1137. doi:10.4018/978-1-59904-941-0.ch064

28. Dubuisson M-P, Jain AK. A modified Hausdorff distance for object matching. Proc 12th Int Conf Pattern Recognit. 1994;1: 566–568. doi:10.1109/ICPR.1994.576361

29. Burnett M. Blocking Brute Force Attacks. UVA Comput Sci. 2007;

30. Zou H, Hastie T. Regularization and variable selection via the elastic net. J R Stat Soc Ser B Stat Methodol. 2005;67: 301–320. doi:10.1111/j.1467-9868.2005.00527.x

31. Pedregosa F, Varoquaux G, Gramfort A, Michel V, Thirion B, Grisel O, et al. Scikit-learn: Machine learning in Python. J Mach Learn Res. 2011;12: 2825–2830.

32. Tibshirani R. Regression Shrinkage and Selection Via the Lasso. J R Stat Soc Ser B. 1996;58: 267–288. doi:10.1111/j.2517-6161.1996.tb02080.x

33. Ho TK. Random decision forests. Proceedings of the International Conference on Document Analysis and Recognition, ICDAR. 1995. pp. 278–282. doi:10.1109/ICDAR.1995.598994

34. Madsen H, Thyregod P. A Generalized linear Model with binomial distribution and probit link function has been used as classifier. Introduction to General and Generalized Linear Models. Chapman & Hall/CRC; 2011.

35. Kira K, Rendell LA. Feature selection problem: traditional methods and a new algorithm. Proceedings Tenth National Conference on Artificial Intelligence. 1992.

36. Xuan G, Zhu X, Chai P, Zhang Z, Shi YQ, Fu D. Feature selection based on the Bhattacharyya distance. Proceedings - International Conference on Pattern Recognition. 2006. doi:10.1109/ICPR.2006.558

37. Keinosuke Fukunaga. Introduction to statistical pattern recognition 2nd edition. Academic Press. 1990. doi:10.1016/0098-3004(96)00017-9

38. Christin C, Hoefsloot HCJ, Smilde AK, Hoekman B, Suits F, Bischoff R, et al. A critical assessment of feature selection methods for biomarker discovery in clinical proteomics. Mol Cell Proteomics. 2013;12: 263–276. doi:10.1074/mcp.M112.022566

39. Haykin S. Neural networks: a comprehensive foundation. The Knowledge Engineering Review. 1994. doi:10.1017/S0269888998214044

40. Gorban AN, Zinovyev AY. Principal Graphs and Manifolds. Handbook of Research on Machine Learning Applications and Trends: Algorithms, Methods and Techniques. Information Science Reference; 2009. pp. 28–59.

41. Shimizu K, Short DA, Kedem B. Single- and Double-Threshold Methods for Estimating the Variance of Area Rain Rate. J Meteorol. 1993;71.

42. Kohavi R. A study of cross-validation and bootstrap for accuracy estimation and model selection. Proc 14th Int Jt Conf Artif Intell - Vol 2. 1995;2: 1137–1143. doi:10.1067/mod.2000.109031

43. BIPM. Guides in metrology, Guide to the Expression of Uncertainty in Measurement (GUM) and International Vocabulary of Metrology (VIM). 2008.

44. Berriz GF, Beaver JE, Cenik C, Tasan M, Roth FP. Next generation software for functional trend analysis. Bioinformatics. 2009;25: 3043–3044. doi:10.1093/bioinformatics/btp498

45. Patel VB, Wang Z, Fan D, Zhabyeyev P, Basu R, Das SK, et al. Loss of p47phox subunit enhances susceptibility to biomechanical stress and heart failure because of dysregulation of cortactin and actin filaments. Circ Res. 2013;112: 1542–56. doi:10.1161/CIRCRESAHA.111.300299

46. Karsanov N V., Pirtskhalaishvili MP, Semerikova VJ, Losaberidze NS. Thin myofilament proteins in norm and heart failure I. Polymerizability of myocardial Straub actin in acute and chronic heart failure. Basic Res Cardiol. 1986;81: 199–212. doi:10.1007/BF01907384

47. Childers RC, Sunyecz I, West TA, Cismowski MJ, Lucchesi PA, Gooch KJ. Role of the Cytoskeleton in the Development of a Hypofibrotic Cardiac Fibroblast Phenotype in Volume Overload Heart Failure. Am J Physiol Heart Circ Physiol. 2018;316: H596–H608. doi:10.1152/ajpheart.00095.2018

48. Liu YH, Yang XP, Sharov VG, Nass O, Sabbah HN, Peterson E, et al. Effects of angiotensin-converting enzyme inhibitors and angiotensin II type 1 receptor antagonists in rats with heart failure: Role of kinins and angiotensin II type 2 receptors. J Clin Invest. 1997;99: 1926–35. doi:10.1172/JCI119360

49. Schrier RW, Abdallah JG, Weinberger HHD, Abraham WT. Therapy of heart failure. Kidney Int. 2000;57: 1418–25. doi:10.1046/j.1523-1755.2000.00986.x

50. Aoyagi T, Matsui T. Phosphoinositide-3 kinase signaling in cardiac hypertrophy and heart failure. Curr Pharm Des. 2011;17: 1818–24.

51. Ennis I, Aiello E, Cingolani H, Perez N. The Autocrine/Paracrine Loop After Myocardial Stretch: Mineralocorticoid Receptor Activation. Curr Cardiol Rev. 2013;9: 230–40. doi:10.2174/1573403x113099990034

52. Sullivan RKP, WoldeMussie E, Pow D V. Dendritic and synaptic plasticity of neurons in the human age-related macular degeneration retina. Investig Ophthalmol Vis Sci. 2007;48: 2782–91. doi:10.1167/iovs.06-1283

53. Sohn YI, Lee NJ, Chung A, Saavedra JM, Scott Turner R, Pak DTS, et al. Antihypertensive drug Valsartan promotes dendritic spine density by altering AMPA receptor trafficking. Biochem Biophys Res Commun. 2013;439: 464–70. doi:10.1016/j.bbrc.2013.08.091

54. Frank RN. Growth factors in age-related macular degeneration: Pathogenic and therapeutic implications. Ophthalmic Res. 1997;29: 341–53. doi:10.1159/000268032

55. Glenn J V., Stitt AW. The role of advanced glycation end products in retinal ageing and disease. Biochim Biophys Acta - Gen Subj. 2009;1790: 1109–16. doi:10.1016/j.bbagen.2009.04.016

56. Grossniklaus HE, Green WR. Choroidal neovascularization. Am J Ophthalmol. 2004;137: 496–503. doi:10.1016/j.ajo.2003.09.042

57. Yuan X, Gu X, Crabb JS, Yue X, Shadrach K, Hollyfield JG, et al. Quantitative Proteomics: Comparison of the Macular Bruch Membrane/Choroid Complex from Age-related Macular Degeneration and Normal Eyes. Mol Cell Proteomics. 2010;9: 1031–46. doi:10.1074/mcp.m900523-mcp200

58. Lee AY, Kulkarni M, Fang AM, Edelstein S, Osborn MP, Brantley MA. The effect of genetic variants in SERPING1 on the risk of neovascular age-related macular degeneration. Br J Ophthalmol. 2010;94: 915–7. doi:10.1136/bjo.2009.172007

59. Higgins P. Balancing AhR-Dependent Pro-Oxidant and Nrf2-Responsive Anti-Oxidant Pathways in Age-Related Retinopathy: Is SERPINE1 Expression a Therapeutic Target in Disease Onset and Progression? J Mol Genet Med. 2015;8: 101. doi:10.4172/1747-0862.1000101

60. Miyata M, Ikeda Y, Nakamura S, Sasaki T, Abe S, Minagoe S, et al. Effects of Valsartan on Fibrinolysis in Hypertensive Patients With Metabolic Syndrome. Circ J. 2012;76: 843–51. doi:10.1253/circj.cj-12-0153

61. Oubiña MP, De las Heras N, Vázquez-Pérez S, Cediel E, Sanz-Rosa D, Ruilope LM, et al. Valsartan improves fibrinolytic balance in atherosclerotic rabbits. J Hypertens. 2002;20: 303–10. doi:10.1097/00004872-200202000-00021

62. Albert-Fort M, Hombrebueno JR, Pons-Vazquez S, Sanz-Gonzalez S, Diaz-Llopis M, Pinazo-Durán MD. Retinal neurodegenerative changes in the adult insulin receptor substrate-2 deficient mouse. Exp Eye Res. 2014;124: 1–10. doi:10.1016/j.exer.2014.04.018

63. Zhang R, Liu Z, Zhang H, Zhang Y, Lin D. The COX-2-selective antagonist (NS-398) inhibits choroidal neovascularization and subretinal fibrosis. PLoS One. 2016;11: e0146808. doi:10.1371/journal.pone.0146808

64. Wang X, Ma W, Han S, Meng Z, Zhao L, Yin Y, et al. TGF-β participates choroid neovascularization through Smad2/3-VEGF/TNF-α signaling in mice with Laser-induced wet age-related macular degeneration. Sci Rep. 2017;7: 9672. doi:10.1038/s41598-017-10124-4

65. Skeie JM, Zeng S, Faidley EA, Mullins RF. Angiogenin in age-related macular degeneration. Mol Vis. 2011;17: 576–82.

66. Aguirre-Plans J, Piñero J, Sanz F, Furlong LI, Fernandez-Fuentes N, Oliva B, et al. GUILDify v2.0: A Tool to Identify Molecular Networks Underlying Human Diseases, Their Comorbidities and Their Druggable Targets. J Mol Biol. 2019; 30117–2. doi:10.1016/j.jmb.2019.02.027

67. Hegab Z, Gibbons S, Neyses L, Mamas M. Role of advanced glycation end products in cardiovascular disease. World J Cardiol. 2012;4: 90–102. doi:10.4330/wjc.v4.i4.90

68. Banevicius M, Vilkeviciute A, Kriauciuniene L, Liutkeviciene R, Deltuva VP. The Association Between Variants of Receptor for Advanced Glycation End Products (RAGE) Gene Polymorphisms and Age-Related Macular Degeneration. Med Sci Monit. 2018;24: 190–199. doi:10.12659/msm.905311

69. Pickering RJ, Tikellis C, Rosado CJ, Tsorotes D, Dimitropoulos A, Smith M, et al. Transactivation of RAGE mediates angiotensin-induced inflammation and atherogenesis. J Clin Invest. 2019;129: 406–421. doi:10.1172/JCI99987

70. Garbayo E, Gavira JJ, De Yebenes MG, Pelacho B, Abizanda G, Lana H, et al. Catheter-based intramyocardial injection of FGF1 or NRG1-loaded MPs improves cardiac function in a preclinical model of ischemia-reperfusion. Sci Rep. 2016;6: 25932. doi:10.1038/srep25932

71. Lakó-Futó Z, Szokodi I, Sármán B, Földes G, Tokola H, Ilves M, et al. Evidence for a Functional Role of Angiotensin II Type 2 Receptor in the Cardiac Hypertrophic Process in Vivo in the Rat Heart. Circulation. 2003;108: 2414–22. doi:10.1161/01.CIR.0000093193.63314.D9

72. Galindo CL, Ryzhov S, Sawyer DB. Neuregulin as a heart failure therapy and mediator of reverse remodeling. Curr Heart Fail Rep. 2014;11: 40–9. doi:10.1007/s11897-013-0176-2

73. Xu J, De Winter F, Farrokhi C, Rockenstein E, Mante M, Adame A, et al. Neuregulin 1 improves cognitive deficits and neuropathology in an Alzheimer’s disease model. Sci Rep. 2016;6: 31692. doi:10.1038/srep31692

74. Kaarniranta K, Salminen A, Haapasalo A, Soininen H, Hiltunen M. Age-related macular degeneration (AMD): Alzheimer’s disease in the eye? J Alzheimer’s Dis. 2011;24: 615–31. doi:10.3233/JAD-2011-101908

75. Verweij N, Eppinga RN, Hagemeijer Y, Van Der Harst P. Identification of 15 novel risk loci for coronary artery disease and genetic risk of recurrent events, atrial fibrillation and heart failure. Sci Rep. 2017;7: 2761. doi:10.1038/s41598-017-03062-8

76. Kaarniranta K, Sinha D, Blasiak J, Kauppinen A, Veréb Z, Salminen A, et al. Autophagy and heterophagy dysregulation leads to retinal pigment epithelium dysfunction and development of age-related macular degeneration. Autophagy. 2013;9: 973–84. doi:10.4161/auto.24546

77. Kawano H, Cody RJ, Graf K, Goetze S, Kawano Y, Schnee J, et al. Angiotensin II Enhances Integrin and α-Actinin Expression in Adult Rat Cardiac Fibroblasts. Hypertension. 2012;35: 273–9. doi:10.1161/01.hyp.35.1.273

78. Bujak M, Frangogiannis NG. The role of IL-1 in the pathogenesis of heart disease. Arch Immunol Ther Exp (Warsz). 2009;57: 165–76. doi:10.1007/s00005-009-0024-y

79. Turner NA. Effects of interleukin-1 on cardiac fibroblast function: Relevance to post-myocardial infarction remodelling. Vascul Pharmacol. 2014;60: 1–7. doi:10.1016/j.vph.2013.06.002

80. Nassar K, Grisanti S, Elfar E, Lüke J, Lüke M, Grisanti S. Serum cytokines as biomarkers for age-related macular degeneration. Graefe’s Arch Clin Exp Ophthalmol. 2015;253: 699–704. doi:10.1007/s00417-014-2738-8

81. Zhang YN, Vernooij F, Ibrahim I, Ooi S, Gijsberts CM, Schoneveld AH, et al. Extracellular vesicle proteins associated with systemic vascular events correlate with heart failure: An observational study in a dyspnoea cohort. PLoS One. 2016;11: e0148073. doi:10.1371/journal.pone.0148073

82. Zaman AKMT, French CJ, Schneider DJ, Sobel BE. A Profibrotic Effect of Plasminogen Activator Inhibitor Type-1 (PAI-1) in the Heart. Exp Biol Med. 2009;234: 246–54. doi:10.3181/0811-rm-321

83. Messaoudi S, Azibani F, Delcayre C, Jaisser F. Aldosterone, mineralocorticoid receptor, and heart failure. Mol Cell Endocrinol. 2012;350: 266–72. doi:10.1016/j.mce.2011.06.038

84. Chakravarthy U, Wong TY, Fletcher A, Piault E, Evans C, Zlateva G, et al. Clinical risk factors for age-related macular degeneration: A systematic review and meta-analysis. BMC Ophthalmol. 2010;10: 31. doi:10.1186/1471-2415-10-31
