# Supplementary material for: In-silico simulated prototype-patients using TPMS technology to study a potential adverse effect of sacubitril and valsartan
Source: PLoS One. 2020 Feb 13;15(2):e0228926. doi: 10.1371/journal.pone.0228926 (PMC7018085; doi:10.1371/journal.pone.0228926)
Supplement: Attachment — Submitted filename: rebuttal_letter_.docx [file pone.0228926.s002.docx]

Barcelona, December 19^th^, 2019

Dear Editor,

We wish to thank you for giving us the possibility of revising the text (manuscript reference PONE-D-19-21700). We also want to thank the anonymous reviewers for their critical evaluation and constructive feedback. Please, find our answers to the reviewers’ comments and the modified manuscript files enclosed. We have also modified the title of the manuscript to clarify that the objective of this work is a theoretical, not clinical, study. Modifications to the last submitted version of the manuscript are highlighted in yellow background. In the present review, we have reinforced an explanation of the method and remarked the theoretical nature of the case study. We hope the major amendments herein introduced will make the current manuscript version suitable for publication. In this letter, we also include the Funding Statement and Competing Interests Statement as requested.

Yours sincerely,

José Manuel Mas and Baldo Oliva

Anaxomics Biotech SL

Structural Bioinformatics Group (Universitat Pompeu Fabra)

**Funding Statement**

GJ, VJ, CSV, JLR, AP and JMM have commercial affiliation to Anaxomics Biotech SL. The funders provided support in the form of salaries for authors JAP, NFF, BO, GJ and VJ, but did not have any additional role in the study design, data collection and analysis, decision to publish, or preparation of the manuscript. The specific roles of these authors are articulated in the ‘author contributions’ section.

**Competing Interests Statement**

The commercial affiliation of the authors does not alter our adherence to PLOS ONE policies on sharing data and materials

**REVIEW COMMENTS TO THE AUTHOR**

***Reviewer #1****:The Authors present MD as "a common/recurrent adverse effect" of therapy with sacubitril valsartan, which does not seem to be the case. Indeed, 5 years after the publication of the PARADIGM HF study, 3 years after the latest ESC guidelines and 2 years after the update to ACC/AHA guidelines, a dedicated literature search does not give any results except for some outdated concerns of a greater risk of Alzheimer's disease (AD) and MD based on conceptual considerations. See for example doi 10.1038/nrcardio.2016.200: "Additionally, inhibition of neprilysin metabolism of amyloid-β peptides might have an effect on Alzheimer disease, age-related macular degeneration, and cerebral amyloid angiopathy". Nonetheless, the increased risk of AD has not been confirmed by dedicated studies. Therefore, the Authors propose a sophisticated approach inevitably burdened by a lot of assumptions and simplifications to solve a problem (i.e., how to predict MD) that does not seem to exist.*

We would like to thank the reviewer for such a valuable critic from the point of view of a cardiologist. As the reviewer pointed out, it was not correct to present macular degeneration as a “common/recurrent adverse effect”, as there are no clear evidences. We have reoriented the manuscript, because it was not in our interest to point on the adverse effects but on the potentiality of our approach to help with mechanistical proposals that can explain potential effects. Consequently, it is more appropriate to focus on a “potential adverse effect” and we have modified the Abstract and the manuscript accordingly:

We use TPMS to generate a range of mechanism of action models explaining the relationship between sacubitril/valsartan and heart failure (the indication), as well as evaluating their association with macular degeneration (a potential adverse effect).

As the reviewer indicates, the two last clinical trials evaluating sacubitril/valsartan (PARADIGM-HF and PARAGON-HF) did not find evidences of macular degeneration as a side effect of the treatment. However, we do think that there are hints indicating that macular degeneration could be a potential long-term effect, and we insist that this is just “potential” and it has been the main reason for the use of our programs which try to infer the causes. Here we hope to answer the reviewer by pointing out many of the hints on which the hypothesis is based. These hints are founded on the inhibition of neprilysin by sacubitril. As neprilysin degradates amyloid-β in the brain, its inhibition could cause the accumulation of amyloid-β in a long term, leading to diseases like macular degeneration. This issue was already raised in the last ESC guidelines on Acute and Chronic Heart Failure:

“*There are additional concerns about its effects on the degradation of beta-amyloid peptide in the brain, which could theoretically accelerate amyloid deposition. However, a recent small 14-day study with healthy subjects showed elevation of the beta-amyloid protein in the soluble rather than the aggregable form, which if confirmed over longer time periods in patients with HFrEF may indicate the cerebral safety of sacubitril/valsartan. Long-term safety needs to be addressed.*”

The same issue had also been highlighted in some publications. For instance, *Feldman et al. (2016)* [1] associates directly this possibility with macular degeneration:

“*Studies in the eye suggest that a similar pathogenic mechanism may contribute to the development of age-related macular degeneration, the most common cause of legal blindness among persons older than 50 years in the United States.*”

…

“With respect to ocular risks, neprilysin-deficient mice developed retinal pigment epithelial cell degeneration and subretinal deposits similar to those found in humans with age-related macular degeneration”

*Zhang et al. (2018)* [2] also relates the aggregation of amyloid-β in the brain with a potential development of age-related macular degeneration:

“*For example, as an off-target effect, inhibition of NEP could increase levels of amyloid beta in brain and eyes leading to development of symptomatic Alzheimer’s disease or age-related macular degeneration, respectively and in turn increase the risks of cognitive and/or visual dysfunction that need to be monitored closely when using a NEP inhibitor.*”

Additionally, as we point out in the text, the current PERSPECTIVE clinical trial pretends to study the cognitive function of sacubitril/valsartan precisely to clarify this issue. All these references have been cited in the Introduction of the manuscript to justify why we have chosen this particular case study:

However, several publications warned about the potential long-term negative implications of using a neprilysin inhibitor like sacubitril [15-19]. Neprilysin plays a critical role at maintaining the amyloid-β homeostasis in the brain, and the alteration of amyloid-β levels has been linked to a potential long-term development of Alzheimer’s disease or Macular Degeneration (MD) [15,17,19–21]. During the clinical trials PARADIGM-HF and PARAGON-HF with sacubitril/valsartan no serious effects were detected [14,22]. Still, their patient follow-up was relatively short and not specialized in finding neurodegenerative specific symptoms. For this reason, in a forthcoming PERSPECTIVE trial (NCT02884206) a battery of cognitive tests was taken [18]. In line with this, the application of systems biology methods may shed light to the potential relationship between the treatment and the adverse effect

We agree with the reviewer and wish to remark that the present study is just a theoretical example of how to use TPMS to analyze the potential responses of a treatment. We think that making such systems-biology analysis in parallel with current clinical trials can help to understand better the potential relationships between a treatment and potential (or observed) adverse effects. We have reinforced in the text that the outcomes of the program are theoretical and may not reflect known clinical outcomes. In order to highlight the theoretical nature of the study, we have rewritten the introduction accordingly weighting more on the method than in the case study.

***Reviewer #2****: Jorba et al., used the Therapeutic Performance Mapping System (TPMS) approach to look for biomarkers which can predict MD in HF patients treated with sacubitril/valsartan. The potential for in-silico clinical trials is clearly shown by this system biology approach. There is a strong methodological base to test the hypothesis on. Although it is based on a string of assumptions inherent to the methodology, which make the eventual translation difficult. At some points the manuscript seems a bit tedious, and some analyses seem to be redundant.*

We thank the reviewer for his/her insightful assessment and critical feedback of the manuscript. We agree that some parts need to be explained more clearly to make the manuscript less tedious. We carefully proofread the manuscript again, modifying the parts that were difficult to understand. For instance, too long sentences have been rewritten to facilitate their understanding. Also, the results section has been restructured to make the reading less tedious. We hope that these modifications improve the reading of our manuscript.

***Major***

1- *The approach depends heavily on assumptions and definitions. For example, the HPN is created based on proteins related to the disease (heart failure in this case) from BED. It is difficult to retrieve these proteins used as input, however this input of proteins determines strongly all the other analyses. HF is an extreme heterogeneous disease which has many etiologies and a diverse scale on pathomechanisms. For example: is the input from BED mainly based on ischemic HF, or HF due to abnormal loading conditions?*

We agree with the reviewer to clarify the text. Accordingly, we have modified the text both in the manuscript and supplementary material. First, the HPN is not built from the HF effectors of the BED, although they do have an impact at generating the final models. As explained in the section “*2.1. Building the Human protein network (HPN)*” of the supplementary material, we build the HPN from the integration of protein-protein interactions of different databases (KEGG, BioGRID, IntAct, REACTOME, TRRUST, HPRD and scientific literature). We improved the explanation and included it in the manuscript so that it is clearer for the reader:

**2.1. Building the Human protein network (HPN)**

To apply the TPMS approach and create the mathematical models of MoAs, a Human Protein Network (HPN) is needed beforehand. In this study, we used a protein-protein interactions network created from the integration of public and private databases: KEGG [29], BioGRID [30], IntAct [31], REACTOME [32], TRRUST [33], and HPRD [34]. In addition, information extracted from scientific literature, which was manually curated, was also included and used for trimming the network. The resulting HPN considers interactions corresponding to different tissues to take into account the effect of the *Stimulus* in the whole body.

Then, as explained in the section “*2.2. Defining active/inactive nodes*” of the main manuscript and supplementary, we use a set of restrictions from different databases (HPRD, DIP, TRRUS, INTACT, REACTOME, BIOGRID, SIDER, DrugBank) and specific gene expression datasets (*Iborra-Egea et al. (2017)* [1]). Finally, these sets of restrictions, along with the input drug target proteins and the HF effectors, will mold the network until obtaining models with enough accuracy. We have introduced some modifications in section 2.3 to clarify the model (see them highlighted in yellow).

Regarding the molecular basis of BED effectors, we explained the details of the protein selection criteria and the pathophysiological processes selected for HF and MD. The explanations are in the final paragraph of section 1 and subsections 1.1. and 1.2 of the Supplementary Material:

**1. Biological Effectors Database (BED) to molecularly describe specific clinical conditions**

[…]

The methodology used for assigning the protein effectors to each pathology starts by defining the pathophysiological processes (functions) according to the general definitions used by the scientists studying the disease. Then, a review of the most recent, relevant and accepted information in the field is performed through PubMed queries, starting from general pathophysiology reviews. An expansion of the effector candidate’s identification is done through reading the relevant original papers from the references or adding searches of important concepts that are not covered enough (molecularly wise) within the reviews read. The final goal of the characterization is to select proteins with an accepted functional role within the disease, and specifically within the functions that define the disease to center the analysis.

**1.1. HF effectors**

Regarding the molecular basis of HF BED proteins, they were characterized as described above and in *Iborra-Egea et al. (2017)* [3]. The definition used of heart failure in the current study has been performed according to the indication of Entresto and to the EMA Assessment report [4]. Thus, it is centered in processes associated to long term changes related to cardiac remodeling (as discussed in the paper were the models were initially presented [3]), that can be cause and consequence of heart failure, not necessarily caused by ischemic causes. The identified functions are detailed in Supplementary Table 12.

**Supplementary Table 12**: Pathophysiological processes present in Heart Failure characterization used in the study.

| **Pathophysiological processes** | **# proteins** |
| --- | --- |
| 1-      Cardiomyocyte cell death (including apoptosis and necrosis) | 46 |
| 2-      Left ventricle extracellular matrix remodelling | 37 |
| 3-      Impaired myocyte contractility | 35 |
| 4-      Hypertrophy | 33 |

**1.2. MD effectors**

MD pathophysiology is tightly related to protein accumulation [5-7]. However, the characterization used for the current study not only included this function, but also other processes associated to MD pathophysiology, including neovascularization, characteristics of wet Age-Related MD and changes associated to geographic atrophy (late stage dry Age-Related MD) [8]. The functions are detailed in Supplementary Table 13.

**Supplementary Table 13**: Pathophysiological processes present in Macular Degeneration characterization used in the study.

| **Pathophysiological processes** | **# proteins** |
| --- | --- |
| 1- Light and oxidative stress: lipid oxidation, lipofuscin, advanced glycation end products (AGEs) | 46 |
| 2- Debris accumulation: Drusen and protein aggregation | 37 |
| 3- Disturbance of lysosomal clearance | 35 |
| 4- Autophagy dysregulation | 33 |
| 5- Immunological processes: chronic inflammation | 46 |
| 6- Mitochondrial defects | 37 |
| 7- Extracellular matrix remodelling and Bruch's membrane thickening | 35 |
| 8- Lipoprotein/lipid metabolism | 33 |
| 9- Retinal cell death | 35 |
| 10- Choroidal neovascularization (wet Age-Related MD) | 33 |

2- *Is tissue-specificity taking into account? Restrictions of the HPN are based on gene expression datasets. However, many proteins/genes involved in the pathogenesis of HF are tissue-specific to the heart. Eventually there is a link in the lowHF and highMD group pointing towards fibrinolysis, how should I interpret these results in the light of tissue specificity?*

We thank the reviewer for raising this interesting point. In this work, the Human Protein Network (HPN) that we have considered is not tissue specific. This is because our objective was to study the effect of a treatment for heart failure (affecting the heart) in a different tissue (macular degeneration affects the eye). However, we do use a gene expression dataset (GSE57345) which is heart-specific and was added as a restriction for the models (i.e to define active/inactive nodes to transfer information along the network). Therefore, the final models will be influenced by this heart-specific dataset. Nevertheless, as the HPN is not tissue-specific, we can still find proteins from other tissues that receive the signal from the treatment. Thus, the relationship between the genes associated with fibrinolysis and macular degeneration is a consequence of considering a general network. We have added a more detailed explanation of the HPN in the main manuscript:

**2.1. Building the Human protein network (HPN)**

To apply the TPMS approach and create the mathematical models of MoAs, a Human Protein Network (HPN) is needed beforehand. In this study, we used a protein-protein interactions network created from the integration of public and private databases: KEGG [29], BioGRID [30], IntAct [31], REACTOME [32], TRRUST [33], and HPRD [34]. In addition, information extracted from scientific literature, which was manually curated, was also included and used for trimming the network. The resulting HPN considers interactions corresponding to different tissues to take into account the effect of the *Stimulus* in the whole body.

We also added a more detailed explanation about the heart-specific restriction in the Supplementary Material:

**2.2. Defining model restrictions**

[...]

Additionally, specific restrictions regarding the phenotype under study can also be used, usually derived from high throughput data or additional protein knowledge.

For this study, we added specific information to our models concerning the changes of gene expression induced by sacubitril/valsartan on HF patients. Specifically, we used the GSE57345 gene expression dataset [26], extracted from GEO database, as in *Iborra-Egea et al. (2017)* [3]. We calculated the expression fold change of genes associated with the HPN and mapped them as activated or inhibited proteins (active if they corresponded to over-expressed genes and inactive -inhibited- for under-expressed).

3- *In line with previous point, in the end a list of biomarkers is proposed, the best-classifier proteins can be used as biomarker. Although no suggestions are made how these should reach clinical implementation. Should these markers be measured in blood, or are they only measurable as RNA in cardiac biopsies?*

TPMS is a theoretical approach to find a molecular explanation linking a treatment (sacubitril/valsartan) and a potential adverse effect (MD). The proposed biomarkers are intended to help to validate the relationship. By no means would the next step after proposing the biomarkers be their direct clinical implementation. The biomarkers proposed would still require an experimental validation before reaching clinical implementation. However, we admit that the title was confusing in this sense. For this reason, we decided to change it, so that the purpose of the manuscript is clearer:

**~~TPMS technology to infer biomarkers of macular degeneration prognosis in~~ *~~in silico~~* ~~simulated prototype-patients under the study of heart failure treatment with sacubitril and valsartan~~**

**In-silico simulated prototype-patients using TPMS technology to study a potential adverse effect of sacubitril and valsartan**

4- *How are the MD effectors determined? Are they also retrieved from BED? The HPN is build upon the BED input from HF. How complete is this one for MD?*

We modified the section “1. *Biological Effectors Database (BED) to molecularly describe specific clinical conditions*” of the Supplementary Material to explain in detail the selection criteria of the protein effectors. In the specific case of MD, the pathophysiological processes considered for the pathology are described below:

**1.2. MD effectors**

MD pathophysiology is tightly related to protein accumulation [5-7]. However, the characterization used for the current study not only included this function, but also other processes associated to MD pathophysiology, including neovascularization, characteristics of wet Age-Related MD and changes associated to geographic atrophy (late stage dry Age-Related MD) [8]. The functions are detailed in Supplementary Table 13.

**Supplementary Table 13**: Pathophysiological processes present in Macular Degeneration characterization used in the study.

| **Pathophysiological processes** | **# proteins** |
| --- | --- |
| 1- Light and oxidative stress: lipid oxidation, lipofuscin, advanced glycation end products (AGEs) | 46 |
| 2- Debris accumulation: Drusen and protein aggregation | 37 |
| 3- Disturbance of lysosomal clearance | 35 |
| 4- Autophagy dysregulation | 33 |
| 5- Immunological processes: chronic inflammation | 46 |
| 6- Mitochondrial defects | 37 |
| 7- Extracellular matrix remodelling and Bruch's membrane thickening | 35 |
| 8- Lipoprotein/lipid metabolism | 33 |
| 9- Retinal cell death | 35 |
| 10- Choroidal neovascularization (wet Age-Related MD) | 33 |

Regarding the last question, the HPN is not built from the HF effectors of the BED, although they have an impact at generating the final models. As explained in the section “**Defining active/inactive nodes** ”, we define the nodes according to gene expression datasets (*Iborra-Egea et al. (2017)* [3]). These definitions, along with the input drug target proteins and the HF effectors, will mold the network until obtaining models with enough accuracy. Therefore, changing the effectors from HF to MD has no impact on network but on the TSignal (i.e. the specific restrictions are changed and affects the signal transmission from input to output). Finally, we have to note that the completeness of the network relies only on the general information stored in databanks and consequently the same biases affect both phenotypes.

5- *It’s difficult to interpret the TSignal values (supplemental figure 1). Low and high is defined as first and fourth quartile. How do the 2nd and 3th quartile fit in supplementary figure 1. How is the distinguishment among the four quartiles, or is there an overlapping spectrum from first to fourth quartile?*

We agree with the reviewer that it is difficult to interpret Supplementary Figure 1 without the 2nd and 3rd quartiles. Following the reviewer’s suggestion, we have remade the Supplementary Figure 1 adding the missing quartiles:

**(a)**	**(b)**

**Supplementary Fig 1:** Histogram of the number of models belonging to High- and Low- (HF in **(a)** and MD in **(b)**) in a range of TSignal values. The models are divided in four quartiles, the 1^st^ and 4^th^ corresponding to the Low- and High- groups for HF and vice versa for MD.

As the formation of the Low- and High- groups is by quartiles, there is no overlap between them. Simply, we order all the Mechanisms of Action by TSignal, and we include the 25% first and last of them in the Low- and High- groups for HF and vice versa for MD. In the figure, it is clear the separation between the first and fourth quartiles in terms of TSignal.

6- *It is a bit confusing that certain aspects seem to intertwine. To me, the most interesting part is the analysis regarding high versus low MD in the lowHF group. However, before this analysis there is a lot of emphasis on the low versus high HF group, which is also interesting, but seems not be the purpose of this manuscript and the further analysis.*

We agree with the reviewer. The most important section in *Results and discussion* is the comparison between MoAs of HF and MD. For this reason, we have deleted the overlapping sentences, rearranged the sections, and added subdivisions:

**1. Stratification of MoAs**

**2. Comparison of MoAs of high/low TSignal associated with HF and MD**

**3. Identification and functional analysis of potential biomarkers**

**3.1. Identification of best-classifier proteins differentiating HF responses**

**3.2. Identification of best-classifier proteins differentiating MD responses**

**3.3. Identification of potential biomarkers differentiating MD responses in Low-HF**

**4. Testing TPMS proposed biomarkers with an alternative approach**

Nevertheless, we kept the complete text of these sections in the **Supplementary Material** for a more detailed explanation.

7- *In addition, it is not clear why the last analysis using GUILDify is performed. Also in the conclusion it is stated that 30 biomarkers are proposed (out of the previous analysis). But thereafter, 10 out of the 30 are proposed to be involved in the comorbidity between HF and MD. What does this mean? The multiple analyses seem to introduce more confusion than clarity at this point.*

We completely agree with the reviewer that the way in which we introduced GUILDify in the manuscript is confusing more than helping. The main purpose of using GUILDify was to confirm the mechanistic role of the 30 proposed biomarkers using a different approach. However, in this section we incorrectly used the term comorbidity, as in our hypothesis, where MD is not caused by HF but by sacubitril/valsartan. For this reason, we decided to remove the confusing parts and make a more understandable explanation of this method.

In the introduction, we changed the following sentence:

Simultaneously, we used GUILDify v2.0 web server [23] as an alternative approach to compare the biomarkers proposed by TPMS and reinforce the results.

In the conclusion, we changed this sentence:

Out of this 30, 10 biomarkers were also found in the alternative, independent molecular context proposed by GUILDify, including some HF and MD effectors such as AGER, NRG1, ITGB5 or IL1A.

Finally, in the fourth section of *Results and discussion*, we made major modifications:

**4. Analysis of proposed biomarkers with GUILDify**

In the previous section, we proposed 30 proteins that could potentially help to identify HF patients at risk of developing MD. To corroborate these biomarkers, we tested how many of them are found using a different approach also based on the use of functional networks. For this purpose, we used GUILDify v2.0 [23], a web server that extends the information of disease-gene associations through the protein-protein interactions network. GUILDify scores proteins according to their proximity with the genes associated with a disease (seeds). Using this web server, we identify a list of top-scoring proteins that are critical on transmitting the perturbation of disease genes through the network. The network used by GUILDify is completely independent from the HPN used in the TPMS, becoming an ideal, independent context to test the potential biomarkers.

Thus, we used GUILDify to indicate which of the potential biomarkers identified by TPMS may have a relevant role in the molecular mechanism of the drug. We ran GUILDify using the two targets of sacubitril/valsartan (NEP, AT1R) as seeds, and selected the top 2% scored nodes (defined as the “top-drug” set). We did the same with the phenotypes of HF and MD, using as seeds the 124 effectors of HF and 163 effectors of MD from the BED database. We merged the top scored sets of HF, MD and top-drug (“top-drug ∪ top-HF ∪ top-MD”) and studied the overlap with the set of 30 biomarkers proposed in the previous section. 10 of the candidate biomarkers are found in the merged set “top-drug ∪ top-HF ∪ top-MD” and are consequently significant (see **Supplementary Tables 10 and 11**).

[...]

According to these findings, the 10 potential biomarkers proposed by TPMS and identified with GUILDify might be prioritized when studying good responder HF patients at risk of MD development.

***Minor***

8- *Try to focus the introduction immediately towards HF instead of cardiovascular diseases in general.*

As we realized we should have given more relevance to the method rather than the pathology, we changed the introduction focusing on systems biology methods applied in clinical trials. However, as suggested, when we introduced sacubitril/valsartan, we focused on HF instead of cardiovascular diseases:

Here, we propose the application of TPMS and protein-network approaches in the specific case study of the drug combination sacubitril/valsartan, used for the treatment of Heart Failure (HF). HF is becoming a major health problem in the western world due to its increasing hospitalization rates [13], with a prevalence being influenced by many factors like age, nutritional habits, lifestyles or genetics. This complicates the development of treatments and the identification of universal biomarkers to stratify the population. To facilitate this segmentation, it is necessary to understand the molecular details of the treatment and the pathology.

9- *Figure 1 (especially a) is difficult to read. I can’t read the text in this figure.*

We would like to apologize for this issue. We have increased the font size of the figure and improved the quality.

10- *In figure 2, how can there be BCP in the upper right corner? What does a differential non-best classifier protein exactly mean?*

We would like to thank the reviewer for such insightful questions. Best-Classifier Proteins (BCP) are the proteins that differentiate best High- and Low- MoAs according to their signal values of the Mechanisms of Action. They are obtained using a Data-Science strategy based on an ensemble of different machine learning algorithms. However, in Fig 2 (renamed as **Fig 3** due to changes in the text) they are represented by their mean signal of the Mechanisms of Action. Proteins in the upper right corner of Fig 3 belong to non-Differential Best-Classifier Proteins. This is so because, even if the mean of the High- and Low- signals is very similar, the machine learning algorithms may be able to differentiate High- and Low- MoAs based on their distribution of signals. Here it is an example of a protein at the upper right corner of Fig 3a: protein P29353, the 181st best protein to distinguish High- and Low- HF Mechanisms of Action (cross-validation AUC = 0.67, P-value = 1.12·10^-4^). P29353 has a Low-HF mean signal of 0.99999999948 and a High-HF mean signal of 0.9999999985. As showed in **Supplementary Fig 4a**, the High- and Low- HF signals are both very close to 1, but if we closely explore the distribution of signals considering all the decimals given by TPMS (**Supplementary Fig 4b**), we can observe a slight differentiation between the two distributions. The machine learning algorithms are able to detect these differences and use this protein as feature to distinguish successfully between High- and Low HF Mechanisms of Action. Nevertheless, the outcome from the machine learning algorithms does not affect the results because we did a double-checking by calculating the *differential Best-Classifier Proteins*. The case of the differential non-Best-Classifier Proteins is answered in the following question. Both cases have been explained in the *Extended version of materials and methods* of the Supplementary Material.

**(a)**	**(b)**

**Supplementary Fig 4:** Histogram of the signal of protein P29353 (non-differential Best-Classifier Protein) in the Mechanisms of Action belonging to High-HF and Low-HF. In **(a)** a general vision of the signal, in **(b)** a detailed vision of the signal.

11- *What does a differential non-BCP imply?*

Differential non-Best-Classifier Proteins are proteins that showed significant signal difference between High- and Low- groups, but that didn’t make the top 200 when filtered by p-value. For example, the protein P40763 is the 241st best feature on distinguishing High- and Low- Heart Failure Mechanisms of Action (cross-validation AUC = 0.66, P-value = 1.22·10^-3^). The distribution of High- and Low-group signals are represented in **Supplementary Fig 5**. In the figure we can appreciate how the distributions of High- and Low- signals are overlapped, complicating their differentiation. Still, the p-value of the cross-validation is below 0.05, reflecting the potential of this feature to differentiate the distinct types of Mechanisms of Action.

**Supplementary Fig 5:** Histogram of the signal of protein P40763 (differential non-Best-Classifier Protein) in the Mechanisms of Action belonging to High-HF and Low-HF.

12- *Are there more proteins which are differential best-classifier proteins which did not reach significance? Ie Opposite effect in low versus high, although not p<0.01.*

Yes indeed, there are 2 Best-Classifier Proteins (P32004, P25100) that are Low-HF+ and High-HF- but have a P-value lower than 0.01. These two Best-Classifier Proteins are not considered differential because their High-HF and Low-HF signal values are too overlapped. However, they are considered Best-Classifier Proteins because they are among the top 200 best features of the machine learning algorithms: P32004 is in position 135, with a cross-validation AUC of 0.69 and cross-validation P-value of 1.43·10^-5^; P25100 is in position 166, with a cross-validation AUC of 0.66 and cross-validation P-value of 4.42·10^-5^.

13- *Input for the GO enriched function LHF+HHF- has only 6 proteins as input, this seems a very low input for a GO enrichment analysis.*

We agree that 6 proteins as input for a functional enrichment analysis seem apparently limited. However, the highlighted output functions have a corrected p-value that is significant by far (<0.00050). Checking in detail the results, we observe how for example we obtain 4 out of 8 genes from the *SCAR complex* function. Taking into account that we are using the Uniprot Swissprot namespace as background genes and that we have only 6 genes, it is enough significant. Here I attach a table with more detailed results of the top 10 enriched functions:

| **# of genes** | **# of total genes** | **Log of odds ratio** | **P-value** | **Adjusted p-value** | **GO term ID** | **Go term name** |
| --- | --- | --- | --- | --- | --- | --- |
| 4 | 8 | 3.89197 | 0 | <0.00050 | GO:0031209 | SCAR complex |
| 3 | 7 | 3.6367 | 0 | <0.00050 | GO:2000601 | positive regulation of Arp2/3 complex-mediated actin nucleation |
| 2 | 5 | 3.49059 | 0 | 0.0015 | GO:0051388 | positive regulation of neurotrophin TRK receptor signaling pathway |
| 3 | 11 | 3.3604 | 0 | <0.00050 | GO:0051386 | regulation of neurotrophin TRK receptor signaling pathway |
| 3 | 13 | 3.26859 | 0 | <0.00050 | GO:0051127 | positive regulation of actin nucleation |
| 3 | 14 | 3.22906 | 0 | <0.00050 | GO:0034315 | regulation of Arp2/3 complex-mediated actin nucleation |
| 2 | 9 | 3.15951 | 0 | 0.0035 | GO:0097484 | dendrite extension |
| 3 | 20 | 3.04658 | 0 | <0.00050 | GO:0016601 | Rac protein signal transduction |
| 3 | 24 | 2.95709 | 0 | <0.00050 | GO:0051125 | regulation of actin nucleation |
| 2 | 17 | 2.84406 | 0.00001 | 0.012 | GO:0032433 | filopodium tip |

14- *The approach with the Hausdorff and Euclidean distances seems a bit redundant as the MDS plot better shows how it ‘actually’ works. Visually, the graph is a bit unattractive, as there is much overlap between the groups, indicating that there is no clear clustering. Maybe the graph has to be split into a high and low HF plot, to better show how the MD groups cluster within the HF groups.*

We agree that it may be redundant. Actually, the idea is to provide two types of approaches that validate the difference observed between the groups. First, a graphical view using the MDS plot, and then using Hausdorff and Euclidean distances to show the numerical results. This is why the numerical results are only displayed on the Supplementary Material.

As advised, we computed the graphs for MD groups within HF groups, which are shown in the **Supplementary Figure 3** and help confirming the results obtained and commented in the discussion. We also introduced the figure in the main manuscript:

Moreover, no clear distinction is found when plotting only the MD MoAs within the Low-HF group (see **Supplementary Fig 3a**). However, regarding the set of High-HF MoAs, we can differentiate two clusters of MoAs: one related to the High-MD group (green crosses); and the other close to MoAs of the Low-MD group (black crosses) (see **Supplementary Fig 3b**).

**(a)**	**(b)**

**Supplementary Fig 3:** **Multidimensional scaling plot of the distances between the Mechanisms of Action of Low/High-Macular Degeneration (MD) and Heart Failure (HF)**. In **(a)** we find the models of both MD groups and Low-HF, whereas in **(b)** the models are for both MD groups and High-HF.

Additionally, we modified the display of Fig 3 (now **Fig 2** due to changes in the text) to make it more eye-friendly:

**Fig 2.** **Multidimensional scaling plot of the distances between the Mechanisms of Action (MoA) of the four groups defined**. Each point represents a MoA. Axes are defined by the most representative dimensions.

15- *The part that describes how biomarkers are selected is to confusing (352-370), try to describe it more compact and to the point.*

As suggested by the reviewer, we tried to make the explanation as simple as possible and added it to the section “3.3. Identification of potential biomarkers differentiating MD responses in Low-HF”:

Because of its clinical relevance, we decided to focus on analyzing the special case of prototype-patients in which the treatment reduces HF (Low-HF) but produces MD adverse effect (High-HF). In order to find these prototype-patients, we: (i) identified 13 Low-HF ∩ Low-MD MoAs and 12 Low-HF ∩ High-MD MoAs; and (ii) compared the protein signal of the two groups and proposed 30 potential biomarkers (**Table 1**). Among the proposed biomarkers, we found 16 proteins active in Low-HF ∩ Low-MD MoAs but inactive in Low-HF ∩ High-MD (15 of them shared with MD best-classifier proteins). On the other hand, 14 proteins were identified as inactive in Low-HF ∩ Low-MD and active in Low-HF ∩ High-MD MoAs (12 of them were MD best-classifier proteins). We calculated the GO enriched functions of these two groups and observed that “phosphatidylinositol bisphosphate kinase activity” is enriched among proteins that are active in Low-HF ∩ Low-MD MoAs. Instead, “fibrinolysis” was found to be enriched among proteins active in Low-HF ∩ High-MD MoAs (**Table 2**). With this, we conclude that among the group of prototype-patients for which sacubitril/valsartan improves HF treatment response, the modulation of fibrinolysis could play a role at inducing the MD adverse effect. Moreover, we propose 12 best-classifier proteins that may be considered as biomarkers for good prognosis of the side effect.

The careful description on how to identify the biomarkers is explained in the materials and methods section 3.3. Potential biomarkers extracted from MoAs.

16- *Line 416-17 “In Fig 2, the differential best-classifier proteins with higher score can be identified by a larger area”. It is not clear what is meant with this sentence?*

In this sentence, we meant that the size of the markers in Fig 2 (now renamed as **Fig 3** due to changes in the text) was proportional to the GUILDify score, but we agree with the reviewer that it was confusing. Especially because when Fig 3 is introduced, GUILDify has not been introduced yet. For this reason, we decided to modify the graph. Now, the size of the markers in Fig 3 is not proportional to the GUILDify score anymore. Instead, it is proportional to the -log(P-value) of the Mann-Whitney U test. Thanks to this modification, Fig 3 is more consistent with the results we discuss in the text.

**Fig 3. Scatter plot of the mean signal values of Low and High-“disease” Mechanisms of Action (MoA).** Scatter plot of the mean signal values of Low-“disease” and High-“disease” MoAs for each protein using as disease Heart Failure (HF) in **(a)** and Macular Degeneration (MD) in **(b)**. The average of the output signal of each protein in High-group is presented versus its value in Low-group. Differential signals (Diff., shown as triangles) are defined as those with opposite sign when comparing High versus Low average, and a p-value < 0.01 when calculating the Mann-Whitney *U* test between the two distributions of signals. Best-classifier proteins (BCP) are colored in red, otherwise they are blue. Sizes of markers are proportional to p-values of the Mann-Whitney *U* test.

17- *Line 462-64 “We found that… to MD”. This sentence does not make sense and is not in line with the rest of the manuscript.*

Following the advice of the reviewer, we have changed the sentence so that it makes sense with the previous statements of the manuscript:

After analyzing the models generated, we found different sets of proteins able to classify the models according to HF treatment efficacy or MD treatment relationship.

**REFERENCES**

1.	Feldman AM, Haller JA, DeKosky ST. Valsartan/sacubitril for heart failure: Reconciling disparities between preclinical and clinical investigations. JAMA - J Am Med Assoc. 2016;315: 25–26. doi:10.1001/jama.2015.17632

2.	Zhang Z lu, Li R, Yang FY, Xi L. Natriuretic peptide family as diagnostic/prognostic biomarker and treatment modality in management of adult and geriatric patients with heart failure: Remaining issues and challenges. J Geriatr Cardiol. 2018;15: 540–546. doi:10.11909/j.issn.1671-5411.2018.08.008

3.	Iborra-Egea O, Gálvez-Montón C, Roura S, Perea-Gil I, Prat-Vidal C, Soler-Botija C, et al. Mechanisms of action of sacubitril/valsartan on cardiac remodeling: a systems biology approach. npj Syst Biol Appl. 2017;3: 1–8. doi:10.1038/s41540-017-0013-4
