# Supplementary material for: In-silico simulated prototype-patients using TPMS technology to study a potential adverse effect of sacubitril and valsartan
Source: PLoS One. 2020 Feb 13;15(2):e0228926. doi: 10.1371/journal.pone.0228926 (PMC7018085; doi:10.1371/journal.pone.0228926)
Supplement: Attachment — Submitted filename: Response to Reviewers.docx [file pone.0228926.s003.docx]

Barcelona, December 19^th^, 2019

Dear Editor,

We wish to thank you for giving us a new opportunity of revising the text (manuscript reference PONE-D-19-21700R1) and apologize if the previous review was not more explicitly highlighting that the approach was theoretical. Please, find our answers to your comments and the modified manuscript files enclosed. Modifications to the last submitted version of the manuscript are highlighted in green background and modifications to the first revision in yellow. In the present review, we have reinforced the theoretical nature of our work that does not involve necessarily clinical effects. We hope the minor amendments herein introduced will make the current manuscript version suitable for publication.

Yours sincerely,

José Manuel Mas and Baldo Oliva

Anaxomics Biotech SL

Structural Bioinformatics Group (Universitat Pompeu Fabra)

**Funding Statement**

GJ, VJ, CSV, JLR, AP and JMM have commercial affiliation to Anaxomics Biotech SL. The funders provided support in the form of salaries for authors JAP, NFF, BO, GJ and VJ, but did not have any additional role in the study design, data collection and analysis, decision to publish, or preparation of the manuscript. The specific roles of these authors are articulated in the ‘author contributions’ section.

**Competing Interests Statement**

The commercial affiliation of the authors does not alter our adherence to PLOS ONE policies on sharing data and materials

**ACADEMIC EDITOR:**

*Both reviewers were satisfied with the reply and the changes made. However, you did not address my comment, which has been as follows:*

*Additionally to the reviewers comments, I would like to ask the authors to more clearly highlight the theoretical nature of their work that addresses mainly the theoretical possibility of the method but not necessarily clinical effects. This is particularly true in view of the absence of evidence that the side effect investigated is relevant in patients. The authors should interpret their findings with these thoughts in mind. Finally, the authors should give some more insight in how they think the method should be used for future research and how this may influence both research and clinical practice.*

*Some of the changes made partly address this but not completely. I would like to ask you to address these points specifically and adjust the manuscript accordingly.*

As the editor suggested, we emphasized more clearly the theoretical nature of our work. We remarked the theoretical nature of the results in both the abstract and the introduction:

All prototype-patients models generated are completely theoretical and therefore they do not necessarily involve clinical effects in real patients.

[…]

In this study, we used TPMS and GUILDify v2.0 to analyze the relationship between sacubitril/valsartan, HF and MD in entirely theoretical models. Because these are theoretical models it is important to note that they are not associated with clinical effects in real patients, they only point on potential mechanisms to explain potential adverse effects.

Additionally, we also highlighted that the prototype-patient models are theoretical in the section “2. Comparison of MoAs with high/low TSignal associated to HF or MD” of the Results and discussion:

Finally, we highlight that, as these distinct groups of prototype-patients are theoretical simulations, they don’t reflect the clinical effects of real patients.

We also remarked the theoretical nature of the biomarkers in the section “3. Identification and functional analysis of potential biomarkers” of the Results and Discussion:

Still, we would like to highlight that the biomarkers have been proposed using a theoretical approach, and that the clinical effects studied may not be present in real patients.

Finally, we included in the Conclusions a discussion about the theoretical nature of the results and the future influence of the method in research and clinical practice:

We notice that the models generated with TPMS are completely theoretical and thus, they are not associated with clinical effects of real patients. Consequently, the biomarkers proposed on the basis of these models are also theoretical and would require an experimental validation. Still, TPMS represents a huge improvement for studying the hypothetical relationship between a drug and an adverse effect. Until now, there were not enough tools that allow to perform an exhaustive study on the MoAs of an adverse effect. Now, with the MoAs and biomarkers proposed by TPMS, we provide the tools for this type of research.
